# How do autistic people fare in adult life and can we predict it from childhood?

**DOI:** 10.1002/aur.2868

**Published:** 2022-12-15

**Authors:** Gordon Forbes, Rachel Kent, Tony Charman, Gillian Baird, Andrew Pickles, Emily Simonoff

**Affiliations:** ^1^ Department of Biostatistics and Health Informatics London, Kings College London Institute of Psychiatry, Psychology and Neuroscience London UK; ^2^ Department of Child and Adolescent Psychiatry, Kings College London Institute of Psychiatry, Psychology and Neuroscience London UK; ^3^ South London and Maudsley NHS Foundation Trust London UK; ^4^ Department of Psychology, Kings College London Institute of Psychiatry, Psychology and Neuroscience London UK; ^5^ Guy's and St Thomas' NHS Foundation Trust London UK; ^6^ NIHR Maudsley Biomedical Research Centre at South London Maudsley NHS Foundation Trust and King's College London UK

**Keywords:** adults, anxiety, children, longitudinal data analysis, psychopathology, risk factors

## Abstract

This study describes social, mental health, and quality of life outcomes in early adulthood, and examines childhood predictors in the Special Needs and Autism Project (SNAP), a longitudinal population‐based cohort. Young autistic adults face variable but often substantial challenges across many areas of life. Prediction of outcomes is important to set expectations and could lead to the development of targeted early intervention. Autistic children were enrolled at age 12 and parents reported outcomes 11 years later when their children were age 23 (*n* = 121). Thirty six percent of autistic adults were in competitive employment or education and 54% had frequent contact with friends. Only 5% of autistic adults were living independently, and 37% required overnight care. Moderate or severe anxiety and depression symptoms were found for 11% and 12% of young adults, respectively. Subjective quality of life was similar to UK averages except for social relationships. Using childhood IQ, autism traits and adaptive functioning meaningful predictions can be made of living situation, employment and education and physical health. Prediction was poor for friendships, mental health outcomes and other aspects of quality of life. Our results suggest that although young autistic adults face challenges across normative, social outcomes, they may be faring better in regard to mental health or quality of life. Childhood IQ, autism traits and adaptive functioning are most useful for predicting outcomes. After accounting for these factors, childhood measurements of behavioral and emotional problems and language offered little improvement in prediction of adult outcomes.

## Lay Summary

We asked parents of 121 autistic people questions about their children at age 12 and again, 11 years later in early adulthood. The autistic young adults faced challenges in employment and education, and many needed support for everyday living. We could make good predictions of these challenges using information from childhood. For other aspects of adult life, including mental health and quality of life, we could not make meaningful predictions of individual difficulties using the information we collected in childhood.

## INTRODUCTION

Knowledge of the challenges and opportunities autistic people face in early adulthood is important to set expectations, plan services, and identify where interventions are needed (Schendel et al., 2022). For predictions to be relevant to an individual, we need to be able to provide a personalized description of what the future may hold, predicting future challenges based on their characteristics and circumstances. Identification of characteristics that predict greater difficulties may allow the development of targeted early intervention and support programs, and be the basis of stratification in future research. We report on a representative cohort of UK autistic children in early adult life, when many of their neurotypical peers would be achieving greater levels of independence. To capture the richness of life we describe the young autistic adults' living situation, employment or education, and friendship as well as subjective reports from parents of mental health and quality of life.

Autistic people commonly face greater challenges than non‐autistic people. Many need extensive support for everyday living, face challenges in employment and education, and have limited friendships or time with peers (Billstedt et al., 2005; Bishop‐Fitzpatrick et al., 2016; Chamak & Bonniau, 2016; Eaves & Ho, 2008; Farley et al., 2009; Gillberg & Steffenburg, 1987; Gillespie‐Lynch et al., 2012; Helles et al., 2017; Howlin et al., 2004; Lord et al., 2020; Magiati et al., 2014; Mason et al., 2021; Steinhausen et al., 2016). Compared to non‐autistic people, autistic adults have been found to have higher rates of anxiety and depression (Croen et al., 2015; Hollocks et al., 2019; Lai et al., 2019) and lower quality of life (Ayres et al., 2018; van Heijst & Geurts, 2015). There is, however, considerable heterogeneity in existing studies. For example, estimates of the proportion of autistic people currently experiencing any anxiety disorder range from 5% to 57% (Hollocks et al., 2019). This has been caused, in part, by previous studies not seeking to recruit samples representative of autistic people. Instead, studies have relied on clinical populations (Eaves & Ho, 2008; Lord et al., 2020; Sevaslidou et al., 2019), or have excluded autistic people with a co‐occurring intellectual disability (Hagberg et al., 2013; Zimmerman et al., 2018). Prior to this study, a single population based cohort has reported outcomes in adulthood. The Yokohama Longitudinal ASD Birth Cohort (Y‐LABiC) (Iwasa et al., 2022) reported outcomes from autistic participants in a Japanese population cohort. While their findings indicated high levels of support needs, low levels of friendship, and challenges in employment and education, these challenges were not as great as had been reported in previous systematic reviews (Mason et al., 2021; Steinhausen et al., 2016).

### Prediction of adult outcomes from childhood

Personalized predictions that guide clinical practice are common in health services. For example, the Q‐risk model for coronary heart disease guides clinical practice and health‐related behavior (Ahmed et al., 2014). There are currently no prognostic models for long‐term outcomes of interest for autistic people. Nonetheless, knowing who may require more support, and the areas of life in which challenges may occur, can help target health, education, and social care services and influence public policy more generally. We address two questions relating to prediction. First, what factors predict adult outcomes? Knowledge of predictors can identify individuals with greatest need and may highlight potential areas for intervention. Secondly, how well can we predict different outcomes? The overall strength of prediction tells us the likelihood that a particular prediction will happen. Even if predictive factors can be identified, if the overall predictive performance is poor, it will be difficult to distinguish between those who may go on to experience different outcomes.

### What is known about childhood predictors of adult social outcomes, mental health and quality of life?

Autism traits and cognitive function are viewed as established predictors of social outcomes in adulthood (Howlin & Magiati, 2017; Lord et al., 2020; Magiati et al., 2014; Seltzer et al., 2004; Taylor & Seltzer, 2010). Lower levels of autism traits and higher cognitive function have been found to be associated with better social outcomes. There is mixed evidence for an association between autism traits or cognitive function with mental health (Kent & Simonoff, 2017; Zimmerman et al., 2018) with both higher and lower levels of autism traits and cognitive ability associated with increased anxiety. Evidence from cross sectional studies of adults suggests that higher levels of cognitive ability are associated with increased depression (Sterling et al., 2008). In adulthood, lower levels of autism traits have been found to predict better quality of life (Mason et al., 2018) but the strength of association from childhood to adulthood is unknown.

Lower adaptive functioning has been found to predict higher levels of anxiety and depression in childhood, but also improvements over time suggesting that by adulthood associations may be weaker (Gotham et al., 2015). This contrasts with positive cross sectional associations between depression symptoms and IQ. Better communication levels have been found to predict employment and independence but these analyses did not adjust for different levels of IQ (Liptak et al., 2011).

Analysese of an autistic sample has found substantial stability in emotional problems, assessed with DSM‐5 diagnosis or symptom counts, between childhood and adolescence (Carter Leno et al., 2022; Hollocks et al., 2022). Over a longer period, using general measures of emotional problems, childhood emotional problems have been found to predict adult emotional problems (Gray et al., 2012; Stringer et al., 2020), but it is not known if general measures of emotional problems in childhood predict specific measures of anxiety and depression. Findings in autistic samples differ from findings in non‐autistic samples where the stability in emotional problems between childhood and adolescence is weaker (Copeland et al., 2013) and weaker associations are observed between childhood and adulthood emotional problems (Kwong, 2019).

Family factors have been found to predict social outcomes, with employment found to be predicted by higher household income, and relationships with friends predicted by better parental support and lower levels of parental education (Chan et al., 2018; Liptak et al., 2011; Wei et al., 2018). Parental mental health has been found to have moderate correlation with the mental health of autistic children (Salazar et al., 2015; Totsika et al., 2011; Weiss et al., 2015; Yorke et al., 2018).

The aims of this study are to describe social outcomes, mental health, and quality of life using data from the Special Needs and Autism Project cohort (SNAP), a population representative sample enrolled at age 12 and followed up 11 years later in early adulthood (Baird et al., 2006). Estimates of the minimum prevalence of autism based on the SNAP sample are 1.2% of the population (Baird et al., 2006). We investigate which late childhood, parent, and family characteristics are predictive of adult outcomes and the degree to which we can predict different outcomes. Finally, we place the outcomes of young adults in this study in the context of the general population, and make comparisons with other cohorts of autistic people, in particular a cohort of autistic people recruited over two decades previously from clinical services in the UK (Howlin et al., 2004).

## METHODS

### Participants

We use data from the Special Needs and Autism Project (SNAP), a longitudinal cohort of autistic people. Children born between July 1990 and December 1991, living in the south‐east of England, were screened for an ICD‐10 diagnosis of childhood autism, Asperger syndrome, pervasive developmental disorder not otherwise specified, or atypical autism (referred to here as ‘autism’ reflecting changes in diagnostic practice) (Baird et al., 2006). Autistic children were identified using clinical records and registers of children with special educational needs. To identify undiagnosed autistic children, screening was carried out on children receiving support in school for learning or behavior problems, and children with any diagnosis relating to speech and communicative impairment. A stratified sample was then assessed for autism by the principal clinical investigators using the Autism diagnostic observation schedule (ADOS‐G) (Lord et al., 2000), Autism Diagnostic Interview ‐ Revised (ADI‐R) (Lord et al., 1994), and interviews with teachers. Full details of the sample selection including a flow chart can be found in (Baird et al., 2006). Children were recruited in late childhood and followed up through adolescence, and into adulthood. Data collection for wave 1 took place between 2001 and 2003. Wave 3 data collection occurred between 2013 and 2015. Participants were eligible for this analysis if they had a completed parent report measure at the third wave of data collection.

Ethical approval for the first wave of data collection was given by the South East Multicentre Research Ethics Committee (REC) (00/01/50). Wave 3 ethical approval was granted by the Camberwell and St. Giles NRES Committee number 12/LO/1770, IRAS project number 112286. Written informed consent was obtained from all participating parents and from the 83 autistic adults with mental capacity to consent; for the remaining 38 participants, who were judged by researchers not to have capacity to consent, a consultee was appointed to determine whether the young adult would wish to participate if he or she had been able to give informed consent.

## MEASUREMENT

### Adult outcome variables

Due to the wide IQ range and communication level of participants many young adults were not able to complete self‐report measures. For adult outcomes we use parent report data, to allow inclusion of participants with all ability levels.

Friendship, employment and education, and living situation were measured using ordinal scales, similar to those used in Howlin et al., 2004. Friendship was defined using four levels, (i) Having a close friend seen more than once a week; (ii) close friends seen at least once every 2 weeks; (iii) Close friends seen less than once every 2 weeks, or time with peers every 2 weeks; (iv) No close friendships and time with peers less than once every 2 weeks. Young adults were classified as having a close friend if, as part of a semi structured interview with parents, it was reported that the young adult had a friendship with an individual which involves shared activities and sharing of confidences. Frequency of contact with friends excluded daytime activities that were organized as part of a person's care.

Employment and education was defined with three levels: (i) competitive employment (either part time of full time) or college university education; (ii) supported/sheltered employment or education; (iii) not in employment, education or training. Participants were classified as being in competitive employment if they worked either full time, or part time (no minimum number of hours) in a job that was not part of a supported or sheltered employment scheme. Education or training included attending mainstream university or further education colleges (providing secondary/post‐age 16 qualifications), or vocational training. Young adults in special educational facilities were classified as receiving supported/sheltered education.

Living situation was defined with five levels (i) living independently; (ii) living with family, with up to 5 hours support; (iii) living with family and requiring over 5 hours support without overnight care, or in residential accommodation with some independence; (iv) Living with family requiring over 10 hours support a week, including overnight care; (v) Specialist residential accommodation with 24 h support.

An overall outcome, defined on a 5‐point Likert scale ranging from “very good” to “very poor,” was defined following the approach of Howlin et al., 2004. The outcome was defined by summing the scores for friendship, living situation, and employment and education. The cut points used when defining the level of overall outcome and further details of outcome definitions are given in Supplementary Material Tables S1–S3.

Quality of life was measured using the four domains of the World Health Organization Quality of Life instrument (WHOQOL‐BREF): physical health, psychological, social relationships, and environment. The WHOQOL‐BREF has been validated for use with autistic adults with a similar factor structure to validations carried out in the general population and good or satisfactory internal consistency (Cronbach's alpha: physical health 0.87, psychological 0.84, social relationships 0.68, environment 0.84 (McConachie et al., 2018)). Details on the items making up each domain are given in Supplementary Material Section S1. Anxiety and depression symptoms were measured using the Beck Anxiety Inventory (BAI) (Beck et al., 1988) and Beck Depression Inventory (BDI) (Aalto et al., 2012). Cut points associated with minimal, medium, mild, and severe symptoms have been established in typically developing populations. The BDI has been validated in an autistic population, with good internal consistency found between items (mean total‐item correlation 0.9) (Williams et al., 2021).

### Childhood predictor variables

Autism traits were measured using the Autism Diagnostic Observation Schedule calibrated severity score (ADOS CSS) (Gotham et al., 2009). Adaptive functioning was measured using parent report of the three subdomains of Vineland Adaptive Behavior Scales (VABS): daily living skills, socialization, and communication (Sparrow & Cicchetti, 1989). Full scale IQ was measured using the Wechsler Intelligence Scale for Children III (WISC‐III) (Wechsler, 1991). For 19 participants unable to access the WISC‐III IQ an IQ score was imputed from other assessments (details given in Supplementary Material Section S2).

Language was measured using Clinical Evaluation of Language Fundamentals (CELF) (Adams & Bigler, 1999) or the Pre‐school CELF (Wiig et al., 1992), depending on language level. To account for participant's varying age we used a development quotient for language in all analysis calculated by dividing language age by actual age. Language age was taken to be the age equivalent score from the respective CELF assessment. For participants who did not have sufficient language to receive a CELF age equivalent score, language age was imputed using the age equivalent score from the VABS communication domain.

Mental health was measured using parental report of the emotional problems, conduct problems, and hyperactivity subscales of the Strengths and Difficulties Questionnaire (SDQ) (Goodman, 1997). In the general population, these domains of the SDQ have been found to have satisfactory internal consistency (Chronbach's alpha: emotional problems 0.67, conduct problems 0.63, hyperactivity 0.77 (Goodman, 2001)).

Maternal mental health was measured by self‐report of the General Health Questionnaire (GHQ‐30) (Goldberg, 1988). Parental education was measured as a binary variable. Neighborhood deprivation was measured using the 2007 Index of multiple deprivation (Noble & Dibben, 2007).

In addition to the assessments at childhood, measures of IQ, autism traits, and behavioral and emotional problems were made at age 16, on a reduced sample, and in adulthood. Longitudinal analysis of trajectories of these can be found in (Simonoff et al., 2019; Stringer et al., 2020).

### Statistical analysis

The analysis was pre‐registered (https://doi.org/10.17605/OSF.IO/48NZ2). Living situation, employment and education, and friendship were modeled using proportional odds models. The domains of quality of life were modeled using linear regression. Due to a skewed distribution, anxiety was modeled using negative binomial regression and depression symptoms were log transformed prior to modeling with linear regression. As autism traits and IQ are viewed as established predictors (Howlin & Magiati, 2017) we investigated non‐linear associators with outcomes by modeling these variables using restricted cubic splines, with three knots in all models (Harrell, 2015).

We group childhood predictors into four sets, loosely based on the likelihood of assessment in clinical practice (Naguy, 2017). The first set consisted of IQ and autism traits. The second set included the three subscales of the Vineland Adaptive Behavior Scales and the language development quotient. Set three, behavior and emotional problems, consisted of the three subscales of the strengths and difficulties questionnaire. Set four, neighborhood and parental characteristics, contained maternal mental health, parental education, and neighborhood deprivation. Predictors were added to models cumulatively in sets (Supplementary Material Table S4). To reduce multiple testing, joint tests were conducted on all variables in each set. Individual predictor variables were only considered statistically significant if the joint test for the predictor set was statistically significant. Sampling weights were used to account for the sampling frame and loss to follow up (Seaman et al., 2012) and multiple imputation was used for missing data. Further details are given in Supplementary Material Section S3.

Optimism corrected predictive performance was calculated using Harrell's bootstrap (50 bootstrap replicates) (Harrell, 2015). Optimism correction estimates model performance for a new observation, not from the existing cohort. A correction is applied to account for models over fitting the data on which they are estimated. The correction for optimism is higher for models including more predictors or worse predictive performance (Riley et al., 2019).

Predictive performance for ordinal outcomes (friendship, living situation and employment and education) was measured using the generalized c‐statistic as conventional r‐squared cannot be calculated from proportional odds models. C‐statistics range from 0.5 to 1 with values above 0.8 generally required for useful individual prediction (Harrell, 2015). For other outcomes, overall performance is measured using r‐squared. For depression, which was log transformed r‐squared was calculated on the transformed scale. *R*‐squared ranges from 0 to 1 (due to optimism correction, negative values can occur when prediction is very poor) values between 0.13 and 0.25 have been considered to show moderate effects and above 0.25 high effects (Cohen, 1992).

## RESULTS

### Sample characteristics

Of the 158 children who completed wave 1 assessments, 121 (77%) completed adult follow up and are included in this analysis. Supplementary Figure S1 shows a flowchart with numbers at the two waves of data collection used in this analysis. Participant characteristics at baseline are given in Table 1. The sample consisted of 106 males (88%). At baseline, median age was 11.6 (Inter quartile range (IQR) 10.9–12.3) and median full‐scale IQ was 71.0 (IQR 56.0–91.0). An unweighted attrition analysis found socio‐economic status and neighborhood deprivation to be associated with loss to follow up from the study (Supplementary material Table S13). These factors are both taken into account in the sampling weights, reducing any bias due to attrition.

**TABLE 1:** Descriptive characteristics of the sample at wave 1 (childhood)

Variable	*N*	Unweighted		Weighted	
		Median (IQR)	Mean (SD)	Median (IQR)	Mean (SD)
Age (years)	121	11.6 (10.9–12.3)	11.7 (0.9)	12.1 (11.1–12.6)	11.9 (0.9)
Male, *n* (%)	121	106 (87.6%)		83%	
Autism traits (ADOS CSS)	121	6 (4–8)	6.1 (2.8)	6 (3–8)	5.6 (3.2)
Full scale IQ	121	71.0 (56.0–91.0)	73.2 (24.4)	69.0 (56.0–90.0)	70.0 (23.4)
Language development	117	54.1 (37.2–70.9)	55.0 (25.2)	53.0 (43.0–64.7)	52.8 (21.7)
Vineland adaptive behavior scales (standard scores)					
VABS—composite	112	47.0 (31.5–57.5)	46.0 (16.8)	54.0 (34.0–61.0)	49.9 (16.0)
VABS—communication	112	56.0 (38.5–73.0)	57.5 (23.9)	59.0 (42.0–79.0)	60.3 (22.0)
VABS—daily living skills	112	44.0 (19.5–59.0)	43.5 (20.4)	58.0 (23.0–66.0)	50.9 (22.4)
VABS—socialization	112	51.0 (43.5–56.0)	49.0 (14.2)	51.0 (48.0–56.0)	50.7 (13.4)
Strengths and difficulties questionnaire					
SDQ total difficulties	114	21.0 (17.0–25.0)	20.9 (5.9)	23.0 (20.0–24.0)	22.1 (4.6)
SDQ conduct problems	114	3.0 (1.0–5.0)	3.4 (2.2)	4.0 (2.0–5.0)	3.9 (2.3)
SDQ ADHD	114	8.0 (6.0–10.0)	7.4 (2.4)	9.0 (7.0–10.0)	7.9 (2.1)
SDQ Emotional problems	114	4.0 (3.0–6.0)	4.6 (2.7)	4.0 (2.0–7.0)	4.5 (2.8)
Parental and neighborhood characteristics					
Parental education—*n* (%)					
Up to high school diploma	121	34 (28.1%)		50%	
Post high school diploma	121	87 (71.9%)		50%	
Maternal mental health (GHQ)	103	2.0 (0.0–8.0)	5.3 (6.7)	1.0 (0.0–7.0)	4.2 (6.0)
Neighborhood deprivation (IMD)	117	12.6 (7.5–19.9)	15.7 (11.2)	18.7 (11.0–20.0)	20.4 (14.1)

*Note*: Weighted summaries are produced using sampling weights which account for loss to follow up and the sampling frame of the study to give estimates of representative of the population the sample was drawn from.

### Outcomes in young adulthood

**FIGURE 1:**
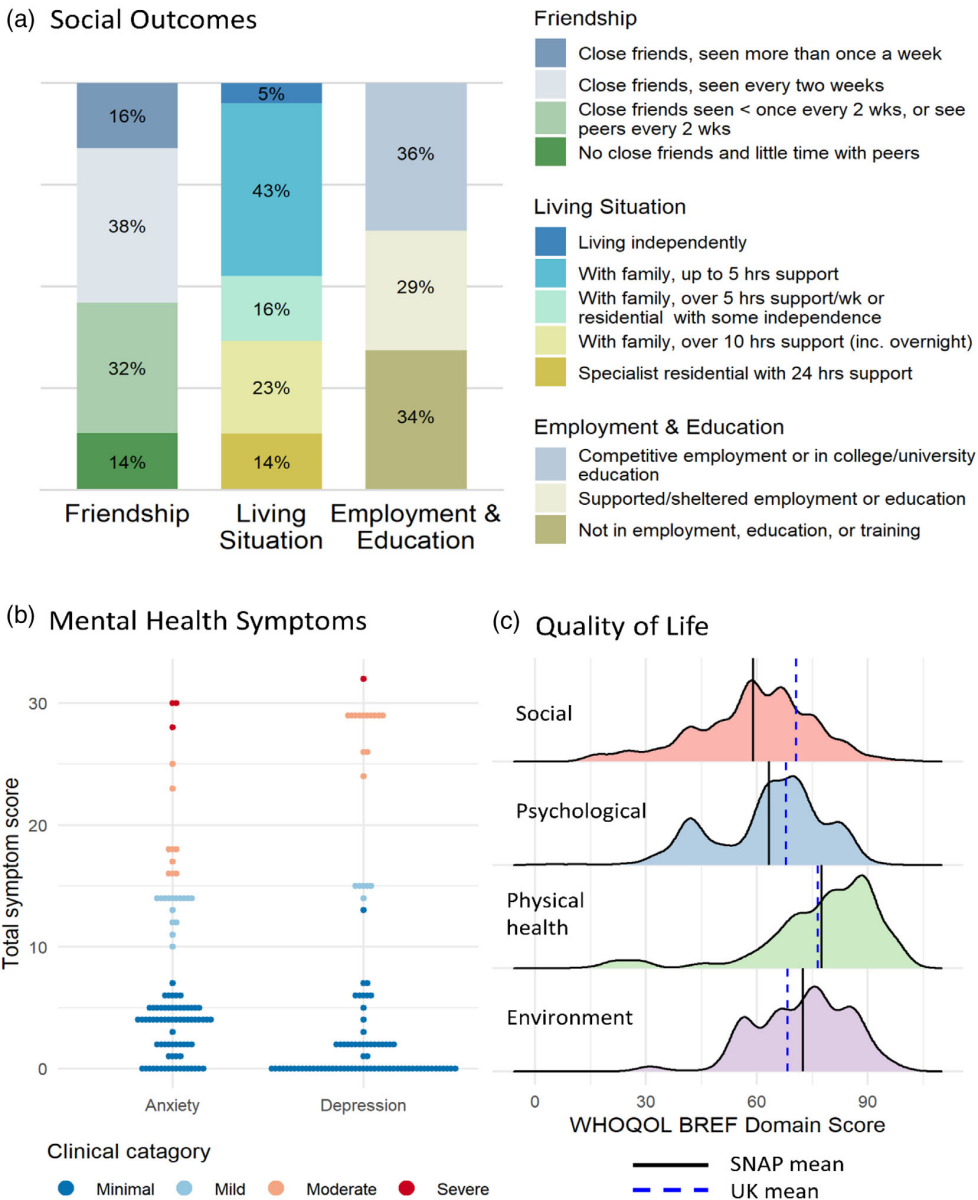
Distributions of outcomes in adulthood after weighting. Plots show the distributions of adult outcomes after weighting. Panel A shows the proportion of participants in each category of the social outcomes, friendship, living situation and Employment and education. Panel B shows the distribution of mental health symptom scores, color coded by clinical cut‐offs. Panel C shows the distribution of quality of life across the four domains with comparisons to UK means.

Figure 1 shows descriptive plots of weighted outcome data. Weighted and unweighted summaries of adult outcomes are given in Table 2. In what follows we report weighted results.

**TABLE 2:** Characteristics of the sample in adulthood

Variable	Unweighted	Weighted
Age (years)—median (IQR) (*N* = 121)	23.0 (22.5–23.7)	22.9 (22.5–23.7)
Friendship—*n* (%) (*N* = 121)		
Close friends, share confidences, see more than once a week	14 (12%)	16%
Close friends, see at least once every 2 weeks outside of activities	39 (32%)	38%
Close friends seen less than once every 2 weeks, or spends time with peers at least once every 2 weeks	31 (26%)	32%
No close friendships and spends time with peers less than once every 2 weeks	37 (31%)	14%
Any close friend—*n* (%) (*N* = 121)		
No close friend	56 (46%)	32%
Any close friend	65 (54%)	68%
Employment and education—*n* (%) (*N* = 121)		
YA employed in competitive employment or in college/university education	42 (35%)	36%
Supported/sheltered employment or education	20 (17%)	29%
Not working or enrolled in education or training	59 (49%)	34%
Living situation—*n* (%) (*N* = 121)		
Living independently	12 (10%)	5%
Living with family, up to 5 h support	42 (35%)	43%
Living with family, support over 5 h/week (w/o overnight care) or residential accomm., some independence	24 (20%)	16%
Living with family and requiring over 10 h support a week from parents (including overnight care)	26 (21%)	23%
Specialist residential accommodation with 24 h support	17 (14%)	14%
Overall outcome—*n* (%) (*N* = 121)		
Very good	22 (18%)	19%
Good	24 (20%)	30%
Fair	43 (36%)	37%
Poor	32 (26%)	15%
Mental health		
Depression (BDI)—mean (SD) (*N* = 108)	5.6 (8.0)	5.7 (9.5)
Depression (BDI) categorical—*n* (%) (*N* = 108)		
Minimal (0–13)	91 (84%)	81%
Mild (14–19)	10 (9%)	7%
Moderate (20–28)	5 (5%)	11%
Severe (29–63)	2 (2%)	1%
Anxiety (BAI)—mean (SD) (*N* = 114)	6.8 (7.8)	6.4 (6.9)
Anxiety (BAI) categorical—*n* (%) (*N* = 114)		
Minimal (0–7)	77 (68%)	73%
Mild (8–15)	20 (18%)	15%
Moderate (16–25)	13 (11%)	9%
Severe (26–63)	4 (4%)	2%
Quality of life: WHOQOL‐BREF		
Physical health—mean (SD) (*N* = 115)	75.7 (16.0)	77.5 (16.3)
Psychological domain—mean (SD) (*N* = 114)	62.2 (15.5)	63.3 (14.7)
Social relationships—mean (SD) (*N* = 113)	56.4 (17.8)	59.0 (15.9)
Environment—mean (SD) (*N* = 114)	71.1 (14.3)	72.5 (12.7)

*Note*: Weighted summaries are produced using sampling weights which take into account loss to follow up and the sampling frame of the study to give estimates of representative of the population the sample was drawn from.

### Social outcomes

Close friendships were reported for 68% percent of autistic adults with approximately half (54%) having frequent contact with close friends (spending time with friends at least once every 2 weeks). This compares to 90% of young adults in the UK who report having at least one close friend (Evans, 2015). Very low levels of social interaction were reported for 14% of young adults in this cohort.

Competitive employment or education was recorded for 36% of young adults, 29% were in supported or sheltered employment, education, or training. Thirty four percent were not in education, employment or training, almost twice the rate of 18% recorded for the 23–24 year olds in the UK population at the time of data collection (Watson, 2021).

High levels of support, including overnight care, was reported for 37% of adults (either with families or in residential accommodation). Moderate levels of independence, either in residential accommodation or living with families was reported for 16% of young adults. 43% of young adults lived with families with low levels (less than 5 h per week) of support. Independent living was rare (5% of young adults). This compares to 54% of the UK population who, at the time of data collection, were living away from home at age 23 (Young Adults Living with Their Parents, 2016).

### Overall outcome

For the overall outcome, 49% of young autistic adults had ‘good’ or ‘very good’ outcomes, 37% had ‘fair’ outcomes, and 15% had ‘poor’ outcomes.

#### Mental health

Parent report depression symptoms were moderate for 11% of young adults, and severe depression was rare (1%). Moderate levels of anxiety were found for 9% of young adults with severe anxiety experienced by 2% of participants. These rates are similar to self‐reported rates from the Avon Longitudinal Study of Parents and Children, a UK general population cohort that reached adulthood at a similar time (Khouja et al., 2019).

#### Quality of life

Mean quality of life was lower in SNAP for the psychological domain (SNAP 63.3 (15.0) vs. UK 67.82 (15.56)) and markedly for social relationships (SNAP 58.8 (15.6) vs. UK 70.52 (20.67)). Quality of life was close to, or higher than previously reported UK general population (Skevington & McCrate, 2012) for physical health (SNAP 77.5, SD (16.3) vs. UK 76.49, SD (16.19)) and environment (SNAP 72.5 (12.5) vs. UK 68.20 (13.81)). In the general population, physical quality of life declines with age (Hawthorne et al., 2006) so while physical quality of life is comparable to a UK average across adulthood, when the age of this cohort is taken into account this represents worse quality of life.

#### Correlations between adult outcomes

Correlations between variables are summarized in Supplementary Material Figures S2–S4. Except for friendships, which was weakly correlated with all other outcomes, outcomes were moderately positively correlated with correlations between 0.22 and 0.75. Correlations were strongest between different measures of mental health symptoms or between different domains of quality of life. Poorer physical health was correlated highly with worse anxiety (correlation 0.63) and lower levels of employment and education (correlation 0.62).

### Childhood predictors of outcomes

**FIGURE 2:**
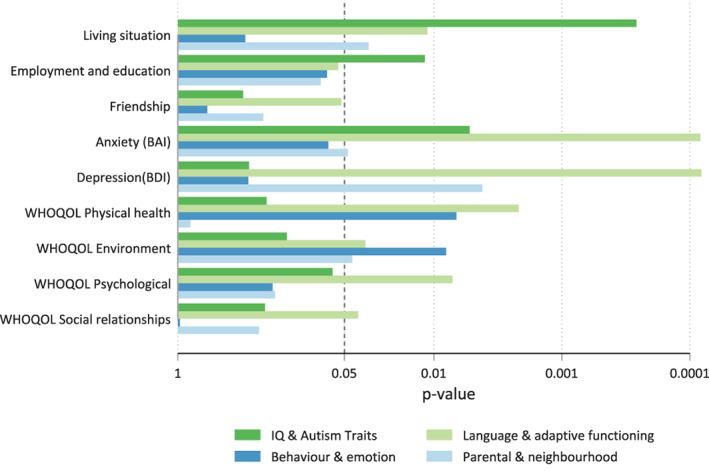
Statistical significance from joint tests of sets of childhood predictors of adult outcomes. *x* axis is reversed scaled so longer bars correspond to smaller *p*‐values.

**FIGURE 3:**
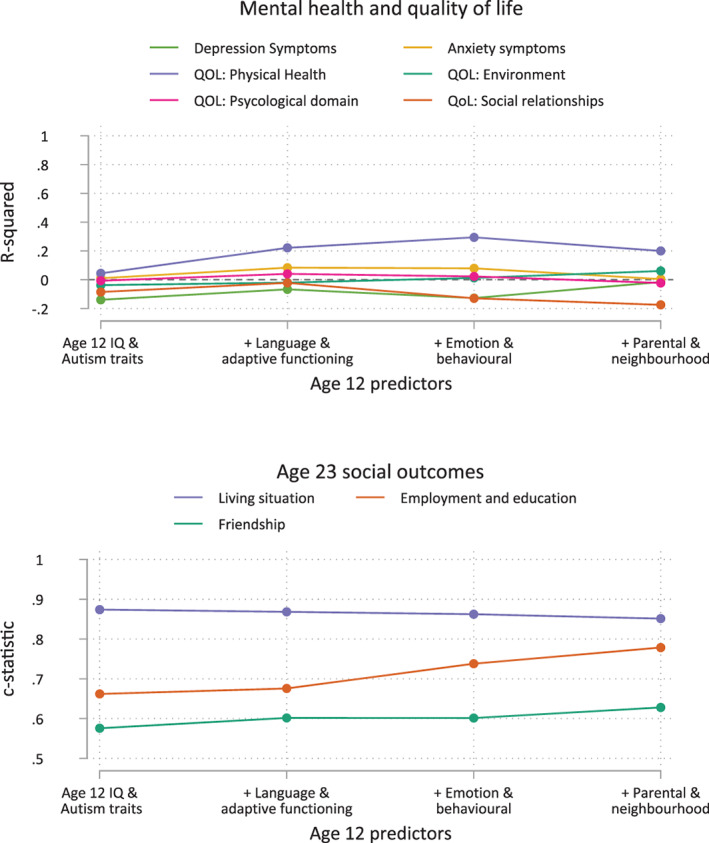
Optimism corrected predictive performance. For all subjective outcomes, except for physical health, *r*‐squared is close to zero indicating that individual prediction is extremely challenging. C‐statistics above, or close to 0.8, indicate good predictive performance for Living situation and Employment and education. Predictors are added cumulatively to the models from left to right.

Table 3 and Figures 2 and 3 show results for the analysis of childhood predictors of adult outcomes. Supplementary Tables S2–S10 give detailed results by outcome.

**TABLE 3:** Optimism corrected performance and statistical significance of predictor sets *p*‐values are from the joint test of new predictor variables added to the model

Outcome	Metric	IQ & Autism Traits	Adaptive functioning & language	Behavioral & emotional problems	Neighborhood characteristics
No. of predictor variables in model		4	8	11	14
Employment and education	C‐statistic	0.66 (*p* = 0.012)	0.68 (*p* = 0.056)	0.74 (*p* = 0.07)	0.78 (*p* = 0.08)
Friendship	C‐statistic	0.58 (*p* = 0.31)	0.60 (*p* = 0.053)	0.60 (*p* = 0.59)	0.63 (*p* = 0.21)
Living situation	C‐statistic	0.87 (*p* < 0.001)	0.87 (*p* = 0.011)	0.86 (*p* = 0.30)	0.85 (*p* = 0.032)
Anxiety (BAI)	Deviance *R* ^2^	0.01 (*p* = 0.005)	0.1 (*p* < 0.001)	0.1 (*p* = 0.07)	0.00 (*p* = 0.047)
Depression (Log BDI)	*R* ^2^	−0.14 (*p* = 0.28)	−0.1 (*p* < 0.001)	−0.1 (*p* = 0.28)	−0.02 (*p* = 0.004)
Physical health	*R* ^2^	0.04 (*p* = 0.20)	0.22 (*p* = 0.002)	0.29 (*p* = 0.007)	0.20 (*p* = 0.80)
Psychological domain	*R* ^2^	−0.01 (*p* = 0.06)	0.04 (*p* = 0.007)	0.02 (*p* = 0.18)	−0.02 (*p* = 0.17)
Social relationships	*R* ^2^	−0.09 (*p* = 0.21)	−0.02 (*p* = 0.04)	−0.13 (*p* = 0.96)	−0.17 (*p* = 0.23)
Environment	*R* ^2^	−0.04 (*p* = 0.14)	−0.02 (*p* = 0.03)	0.01 (*p* = 0.008)	0.06 (*p* = 0.043)

*Note*: The c‐statistic and *R*‐squared are summaries of overall predictive performance of each model. The maximum possible *R*‐squared is 1 with *r*‐squared above 0.25 considered a large overall effect. C‐statistics range from 0.5 to 1 and values above, or close to 0.8, indicate good predictive performance.

#### Social outcomes

Prediction of friendship was poor with no statistically significant predictors. Prediction of employment and education was modest based on IQ and autism traits (c‐statistic 0.66, *p* = 0.12). Despite the joint test for IQ and autism traits being significant (*p* = 0.012) neither were significant on their own so an exploratory analysis of an interaction between the two was conducted. The interaction (*p* = 0.018) showed that the association between IQ and employment education is weak for those high levels of autism traits; for young adults those with lower levels of autism traits higher IQ is associated with greater levels of employment or education (Supplementary Figure S5).

Independence in living situation was predicted well by higher IQs (c‐statistic 0.87, *p* = 0.0003). After adjusting for IQ and autism traits, better VABS daily living skills (*p* = 0.016), better maternal mental health (*p* = 0.034), and higher levels of parental education (*p* = 0.045) were statistically significant predictors although there was no improvement in model performance indicating modest effects.

To illustrate the ability of the models for living situation and employment and education to predict outcomes we present predictions from the models using age 12 IQ and autism traits for children at the upper and lower quartiles for IQ and autism traits. For these outcomes, prediction is possible for all values of prediction and these points are chosen to highlight the ability of the models to differentiate outcomes for children with high and low values of the predictors. The models predict that for children with an IQ of 90 (upper quartile) and ADOS CSS of 3 (lower quartile), 10% will be living independently, 80% will live with family with minimal support and 78% will be in competitive employment or education. A child with an IQ of 57 (lower quartile) and ADOS CSS of 8 (upper quartile) has a 0.1% probability of living independently, a 13% probability of living with family with minimal support, and a 23% probability of being in competitive employment or education. The narrower range in predictions for employment and education compared to living situation reflect the weaker predictive performance of the model for employment and education.

#### Mental health

Overall prediction of anxiety and depression symptoms from childhood variables was very poor with *R*‐squared close to zero or negative in all analysis. Negative *r*‐squared indicates that the optimism correction for increasing the number of variables is greater than the variation in outcome explained. Despite of poor overall performance statistically significant predictors were identified.

Higher childhood IQ (*p* = 0.002), VABS daily living skills (*p* = 0.009), VABS socialization (*p* = 0.004), better maternal mental health (*p* = 0.035), and worse VABS communication (*p* = 0.036) were associated with lower anxiety in adulthood. While the uncorrected test of emotional problems was statistically significant (*p* = 0.023) the joint test (required by our pre‐specified testing strategy) of behavior and emotional problems predicting anxiety was not statistically significant (*p* = 0.07).

VABS better daily living skills (*p* < 0.001), better maternal mental health (*p* = 0.013) and greater neighborhood deprivation (*p* = 0.037) predicted lower levels of depression.

#### Quality of life

Overall prediction of quality of life was poor, except for the physical health domain (maximum *r*‐squared 0.29). Higher VABS daily living skills (*p* = 0.031) and socialization (*p* = 0.004), and poorer VABS communication (*p* = 0.019) predicted better physical health. Adjusting for adaptive functioning, higher levels of conduct problems (*p* = 0.038) and lower levels of emotional problems (*p* = 0.009) predicted better physical health. Prediction of the other domains of quality of life was poor, with *r*‐squared close to zero indicating small effects from statistically significant predictors.

### No association between language and outcomes

Across all outcomes, after adjusting for the three subscales of adaptive functioning, IQ, and autism there was no significant association with language.

## DISCUSSION

### Summary of results

This study provides a multi‐facetted description of life in early adulthood from a representative sample of autistic people enrolled in late childhood and followed up 11 years later as young adults. Follow up occurred prior to the COVID‐19 pandemic. Across objective outcomes, young autistic adults have considerable needs and require support from families or other services. Levels of economic inactivity for autistic adults are double the UK average with many more requiring support in education or employment. One in 20 young autistic adults live independently compared to over half of the general population at this age. The rate of autistic adults with no close friends is three times the rate in the general population. In contrast, subjective, parent report of the young adult's mental health, and psychological and environmental quality of life was similar to the general population. The social and physical health domains of quality of life were lower for autistic adults.

The degree to which outcomes could be predicted from childhood varied. Meaningful individual prediction is possible for living situation and employment and education, and the physical health domain of quality of life. For these outcomes, targeted early intervention may be appropriate, and future intervention trials could stratify by childhood IQ or autism traits, key predictors of outcomes.

While we found statistically significant predictors of levels of friendship, mental health symptoms, and the psychological, social and environmental domains of quality of life, overall prediction was far below the level needed for meaningful individual prediction. This implies that for these important outcomes, the predictive factors used in this study are useful in identifying differences on average, but effect sizes are small, and the amount of variability around these averages is so large that useful individual predictions are not possible. With measures commonly used in research and clinical practice (IQ, ADOS‐CSS, VABS, SDQ) it is, therefore, inappropriate to set expectations of what mental health, or psychological, social or environmental aspects of quality of life in adulthood may be, or target intervention for these outcomes. Challenges in prediction may be due to these outcomes being weakly determined by childhood characteristics or experiences and influenced more strongly by life events, or environmental factors experienced in in adolescence. It may be the case that good mental health, friendships, or quality of life in adulthood are possible for many autistic children. Nonetheless many autistic people may face difficulties across these areas, so interventions to support mental health, quality of life and friendships should be available for all.

Another factor which could have led to difficulties in prediction could poor reliability of parent report measures. With different measurement, or over a shorter time frame better prediction may be possible.

### Results in the context of studies of autistic people

Compared to previous research on autistic people the young adults in this cohort experienced better social outcomes, better mental health, and better quality of life. Using the Howlin definition for an overall social outcome, ‘good’ or ‘very good’ outcomes were recorded for almost half of participants (49%), over double the rate (20%) reported in systematic reviews of studies mostly conducted in clinical populations, or historic cohorts conducted when diagnostic criteria were narrower or when diagnosis were less likely in people without co‐occurring intellectual disabilities (Mason et al., 2021; Steinhausen et al., 2016). The rate of good or very good outcomes in SNAP is more comparable to a recent population based study conducted in Japan (Iwasa et al., 2022) which found 38% of autistic people diagnosed prior to age seven to have ‘very good’ or ‘good’ outcomes. This suggests that while autistic people face considerable difficulties, previous findings may overestimate their challenges.

Social outcomes in SNAP were better than a cohort recruited from clinical services, over two decades earlier (Howlin et al., 2004), in which 22% of young adults had ‘good’ or ‘very good’ outcomes. Follow up for Howlin et al. occurred between 1985 and 1991, a period with similar rates of employment (mean employment over the follow up period 71% for Howlin et al vs. 73% for SNAP) but lower levels of young adults living with parents (37% for Howlin et al. vs. 46% for SNAP). Differences between Howlin et al and SNAP may be due to improvements over time in social outcomes for autistic people or due to differences between who is included in the two samples.

Levels of depression symptoms in this cohort were lower than found in a systematic review, largely based on self‐report or clinical records (Hollocks et al., 2019), although similar to findings in a second review (Lai et al., 2019). Levels of anxiety symptoms are lower than those reported in both the Lai et al. and Hollocks et al. reviews. Lower levels of quality of life have been reported in two recent studies of autistic adults able to self‐report (Mason et al., 2018; Oakley et al., 2021), although a third, finds similar levels (Hong et al., 2016) both from self‐report and parent‐report. One consistency with other studies of quality of life is that the social domain has poorest quality compared to other domains.

There are several explanations for better outcomes in this cohort, compared to other studies of autistic adults. Firstly, the representative nature of the SNAP cohort could lead to better outcomes than in clinic‐based studies, as those with highest needs, or poorest health may be over‐represented in clinical populations. Secondly, there may have been improvements over time in outcomes for autistic people. This could be due to the increasing prevalence of autism leading to different people, with different challenges, being included in research or changes in the way support is provided leading to differences in social outcomes. For example, of the young people seen at follow up, a small number did need highly specialist residential support but none were living in a hospital setting and as a consequence no‐one was classified with a ‘very poor’ overall outcome. A third reason is the use of parent report for subjective outcomes relating to mental health and quality of life. This differs from other research that has been more focused on self‐report or clinical assessments, potentially excluding those who cannot complete self‐report measures (Hollocks et al., 2019; Mason et al., 2018). In the absence of an anxiety diagnosis parents have been found to under‐report anxiety symptoms for autistic children (Kalvin et al., 2020). This may be further exacerbated in adulthood when parents have less contact with their children. Issues of measurement of mental health have focused on anxiety (Kent & Simonoff, 2017), but it is likely that similar issues apply for depression or subjective quality of life. A final factor that could affect mental health outcomes is that for a small number of participants recruited at baseline, mental health difficulties were a barrier to participation in the study in adulthood.

Very good predictions of a young adults living situation and modest predictions of employment and education from late childhood have been reported previously in a cohort conducted in US Early Diagnosis Cohort (Forbes et al., 2021). While in this study we only consider predictions form late childhood, findings from the US early diagnosis cohort indicate that predicting adult outcomes from earlier than this is much more challenging. Consistent with previous research (Howlin & Magiati, 2017), higher IQ predicts greater independence, whereas a combination of higher IQ and lower autism traits predict a higher likelihood of employment and progress in education.

Overall prediction of an individual's mental health in adulthood was very poor, consistent with findings relating to prediction from at least age 12 in the US Early Diagnosis Cohort (Forbes et al., 2021). The findings of weak correlations between childhood emotional problems and adult depression and anxiety (Pearson's correlation 0.21 and 0.17, respectively) and poor predictive performance for these outcomes contrast with the moderate correlation (correlation = 0.43) found between childhood and adult emotional problems domain of the SDQ in a previous analysis of this cohort (Stringer et al., 2020). We see three potential reasons for this difference. Firstly, differences may be due to issues in using parental report for anxiety and depression symptoms. The SDQ has been developed and validated for use as a parent report measure whereas the scales used for anxiety and depression symptoms have not been validated for use as parent report measures. A second reason for the difference may be due to the challenge of relating the more general emotional problems domain of the SDQ to specific measures of anxiety and depression symptoms. The third reason may be that more specific anxiety and depression symptoms may be less amenable to prediction than general measures of emotional problems due to differing developmental trajectories for different areas of mental health (Hollocks et al., 2022).

### Outcome wide trends

After adjusting for IQ and autistic traits, adaptive functioning was a significant predictor of all but two early adult outcomes. This highlights the importance of measuring adaptive function when conducting assessments on autistic children and the potential of adaptive functioning in children to be a target for intervention. Across all outcomes, specific assessments of language contributed very little beyond the prediction possible from IQ, autism traits and adaptive functioning.

### Strengths and limitations

This study uses a population representative cohort, minimizing selection bias. Recruitment to the cohort involved screening for undiagnosed cases of autism improving the representativeness of the cohort by including children yet to be identified by clinical services. Rates of follow up were high and missing data were accounted for in the analysis using weighting and multiple imputation. The analysis followed a pre‐registered plan, and estimates of predictive performance were corrected for the optimism that can occur in small samples. Due to the available sample size, corrections for optimism could mask small effects from predictors, this will particularly be an issue where predictive performance is poor. No correction was made for multiple hypothesis tests so a small number of statistically significant associations could be due to chance.

The use of parent report for outcomes in adulthood provides a consistent rater across the cohort, and avoids those with lower ability levels being excluded from this research, which would be necessary if self‐report was used. Nonetheless challenges in measurement may remain, particularly for mental health symptoms.

## CONCLUSIONS

Many young autistic adults have considerable support needs and face difficulties with education and employment. Levels of friendship and social quality of life are poorer than the general population. Reported outcomes for mental health, and other areas of quality of life appear to be more similar to the general population than has been found in previous research. Levels of independence and employment and education, and to a lesser extent physical health, can be predicted well from late childhood and in these areas it may be possible to develop targeted early intervention or support. Difficulties in adult mental health, quality of life and friendships life are difficult to predict from childhood and, using standard measures, no predictions about adult life from late childhood should be made. Childhood assessment of IQ, autism traits, and adaptive functioning are most important when considering prediction of future outcomes in adulthood. Adaptive functioning was associated with the broadest range of outcomes of any predictors.

## Supporting information


**DATA S1:** Supporting information.

## Data Availability

The data that support the findings of this study are available on request from the corresponding author. The data are not publicly available due to privacy or ethical restrictions.
