# Supplementary material for: How do autistic people fare in adult life and can we predict it from childhood?
Source: Autism Res. 2022 Dec 15;16(2):458–73. doi: 10.1002/aur.2868 (PMC10947100; doi:10.1002/aur.2868)
Supplement: Supplementary file 1 — DATA S1: Supporting information. [file AUR-16-458-s001.docx]

Supplementary material

Contents

[Supplementary Methods 3](#_Toc118373333)

[Section 1: WHOQOL BREF 3](#_Toc118373334)

[Section 2: Additional details on measurement 4](#_Toc118373335)

[Definitions of social outcomes and an overall outcome 4](#_Toc118373336)

[Table S1 - Friendship 4](#_Toc118373337)

[Table S2 – Employment and Education 4](#_Toc118373338)

[Table S3 – Living situation 5](#_Toc118373339)

[Deriving overall classification 5](#_Toc118373340)

[Imputation of childhood IQ for those unable to access the WISC-II 5](#_Toc118373341)

[Section S3: Additional details of Statistical Analysis 6](#_Toc118373342)

[Predictor sets 7](#_Toc118373343)

[Table S4 - Predictor sets used in each analysis 7](#_Toc118373344)

[Supplementary Results 8](#_Toc118373345)

[Figure S1 Flowchart 8](#_Toc118373346)

[Weighted correlations 8](#_Toc118373347)

[Figure S2 - Correlations between age 12 predictors 9](#_Toc118373348)

[Figure S3 - Correlations between age 23 outcomes 10](#_Toc118373349)

[Figure S4 - Correlations between age 12 predictors and age 23 outcomes 11](#_Toc118373350)

[Friendship 12](#_Toc118373351)

[Table S5 - Estimates, p-values for sets are from joint wald tests of all variables in sets. 12](#_Toc118373352)

[Employment and education 13](#_Toc118373353)

[Table S6 - Estimates, p-values for sets are from joint wald tests of all variables in sets. 13](#_Toc118373354)

[Figure S5 – Predicted probability and 95% confidence intervals of competitive employment and education from a model including IQ, ADOS CSS an interaction term between IQ and ADOS CSS at ADOS CSS of 3 and 8. 14](#_Toc118373355)

[Living situation 15](#_Toc118373356)

[Table S6 - Estimates, p-values for sets are from joint wald tests of all variables in sets. 15](#_Toc118373357)

[Physical health 16](#_Toc118373358)

[Table S7 - Estimates, p-values for sets are from joint wald tests of all variables in sets. 16](#_Toc118373359)

[Psychological domain 17](#_Toc118373360)

[Table S8 - Estimates, p-values for sets are from joint wald tests of all variables in sets. 17](#_Toc118373361)

[Social relationships 18](#_Toc118373362)

[Table S9 - Estimates, p-values for sets are from joint wald tests of all variables in sets. 18](#_Toc118373363)

[Environment 19](#_Toc118373364)

[Table S10 - Estimates, p-values for sets are from joint wald tests of all variables in sets. 19](#_Toc118373365)

[Depression symptoms (Log BDI) 20](#_Toc118373366)

[Table S11 - Estimates, p-values for sets are from joint wald tests of all variables in sets. 20](#_Toc118373367)

[Anxiety symptoms 21](#_Toc118373368)

[Table S12 - Estimates, p-values for sets are from joint wald tests of all variables in sets. 21](#_Toc118373369)

[Attrition Analysis 22](#_Toc118373370)

[Table S13 - Attrition analysis 22](#_Toc118373371)

[References 23](#_Toc118373372)

# Supplementary Methods

## Section 1: WHOQOL BREF

The WHOQOL BREF measures subjective quality of life. It is reported as four subscales, physical health, psychological, social, and environment. In this study we report scores transformed onto a 0-100 scale. The items in each subscale relate to:

1. Physical health (7 items)
   1. Physical pain
   2. Medical treatments
   3. Energy for everyday life
   4. Ability to get around
   5. Satisfaction with sleep
   6. Satisfaction with capacity for work
2. Psychological (6 items)
   1. Enjoyment of life
   2. To what extent is your life considered meaningful
   3. Ability to concentrate
   4. Acceptance of bodily appearance
   5. How satisfied you are with yourself
   6. Frequency of negative feelings
3. Social (3 items)
   1. Satisfaction with personel relationships
   2. Satisfaction with support from friends
   3. Satisfaction with sex life
4. Environment (8 items)
   1. How safe do you feel in everyday life
   2. Healthiness of physical environment
   3. Financial situation
   4. Availability of information needed for daily life
   5. Opportunities for leisure activities
   6. Satisfaction with living place
   7. Access to health services
   8. Access to transport

## Section 2: Additional details on measurement

## Definitions of social outcomes and an overall outcome

An ordinal overall outcome measure for autistic adults was detailed by Howlin (Howlin et al., 2004). The outcome consists of three components, friendship, living situation and employment. We define ordinal outcomes for these three components following the approach of Howlin et al, adapting categories where necessary to map to the data collected in SNAP.

### Table S1 - Friendship

| Level | Definition used in SNAP | Definition used in Howlin et al 2004 |
| --- | --- | --- |
| 0 | YA has close friends with whom they share confidences and see more than once a week outside of daytime activities: | Close friendship involving sharing and exchange of confidences and a range of different activities together |
| 1 | YA has close friends who they see at least once every two weeks outside of daytime activities | No details given |
| 2 | YA has close friends who they see less than once every two weeks or report no close friendships but spend time with peers at least once every two weeks | No details given |
| 3 | YA has no close friendships and spends time with peers less than once every two weeks. | no friends; no joint activities. |

### Table S2 – Employment and Education

| Level | SNAP | Definition used in Howlin et al 2004 |
| --- | --- | --- |
| 0 | YA employed in competitive employment or in college/university education | Employed or self-employed; |
| 1 | No data collected | Voluntary work/job training or low-pay scheme; |
| 2 | Supported/sheltered employment or education | Supported/sheltered employment; |
| 3 | Not in work, education, or training | In special center or no occupation. |

### Table S3 – Living situation

| Level | SNAP | Definition used in Howlin et al 2004 |
| --- | --- | --- |
| 0 | Living independently: at university, private rented accommodation | Living independently |
| 1 | Living with family with up to 5 hours support a week from parents | In semi-sheltered accommodation or still at home but with high degree of autonomy |
| 2 | Living with family and requiring support from parents over 5 hours a week (without requiring overnight supervision) or in residential accommodation with some independence | Living with parents, some limited autonomy |
| 3 | Living with family and requiring over 10 hours support a week from parents (including overnight care) | In residential accommodation with some limited autonomy |
| 4 | Specialist residential accommodation with 24 hour support | Residential accommodation with little or no autonomy |
| 5 | Hospital or institution (no one was in this category in SNAP so it was not used). | Hospital or Institution |

### Deriving overall classification

The total of scores for Friendship, Employment and Education, and Living situation are categorized into a 5 level ordinal outcome:

0–2 = Very good outcome

3–4 = Good outcome

5–7 = Fair outcome

8–10 = Poor outcome

11 = Very poor

## Imputation of childhood IQ for those unable to access the WISC-II

IQ was imputed from the British Picture Vocabulary Scale (BPVS) (Dunn et al., 1997), Raven’s Standard Progressive Matrices (SPM) (Raven et al., 1990b) or Raven’s Coloured Progressive Matrices (CPM) (Raven et al., 1990a) using a regression model of the full scale IQ from WISC-III and the BPVS, CPM or SPM. To avoid overly precise imputations which can underestimate the noise in the data a randomly selected residual was added to each estimate.

For a further 10 participants, no direct composite testing was possible and as they had an Adaptive Behaviour Composite score on the VABS below 20 and these cases were assigned an IQ score of 19 to reflect their profound level of intellectual disability.

## Section S3: Additional details of Statistical Analysis

At registration authors had access to the data but no inferential analysis had been conducted relating to this research question. Analysis was implemented with Stata 16 (*Stata Statistical Software: Release 16*, 2019).

**Changes from Pre-specified Analysis.**

Depression symptoms were log transformed prior to modelling with linear regression and r-squared was calculated on the transformed scale. This was changed from the prespecified analysis, which was to use a negative binomial model, as erratic predictive performance indicated severe issues with model fit.

**Multiple Imputation**

Missing data in predictors and outcomes relating to mental health or quality of life was imputed using multiple imputation using chained equations (White et al., 2011), with 25 imputations, and outcomes modelled with predictive mean matching The imputation model included all predictors and outcomes. No imputation was conducted of childhood autism traits and IQ, and adult social outcomes as there was no missing data in those with adult follow up.

**Sampling weights**

Inverse probability weights are used for descriptive statistics and analysis. These adjust for the sampling frame and loss to follow up. Weights are calculated in two stages. Stage 1 weights account for the sampling which was two way stratified based on the Social Communication Questionnaire and the autism diagnosis from local services. Second stage weights account for gender, educational attainment of the parents, neighborhood deprivation and socioeconomic status. Stage 2 do what. To avoid extreme weights, weights are trimmed by replacing weights greater than 3 standard deviations from the mean with the mean plus three standard deviations.

## Predictor sets

For each outcome we run four analysis. In each analysis we include all predictors from the previous analysis, and a new set of predictors. No variable selection is carried out.

### Table S4 - Predictor sets used in each analysis

| **Analysis** | **New predictors** | **Predictors in analysis** |
| --- | --- | --- |
| 1 | Full scale IQ (linear term), Full scale IQ (non-linear term), Autism traits (linear term), Autism traits (non-linear term) | Full scale IQ (linear term), Full scale IQ (non-linear term), Autism traits (linear term), Autism traits (non-linear term) |
| 2 | Language development quotient, VABS - communication, VABS - daily living skills, VABS - socialisation | Full scale IQ (linear term), Full scale IQ (non-linear term), Autism traits (linear term), Autism traits (non-linear term) Language development quotient, VABS - communication, VABS - daily living skills, VABS - socialisation |
| 3 | SDQ conduct problems, SDQ Emotional problems, SDQ ADHD | Full scale IQ (linear term), Full scale IQ (non-linear term), Autism traits (linear term), Autism traits (non-linear term) Language development quotient, VABS - communication, VABS - daily living skills, VABS - socialisation SDQ conduct problems, SDQ Emotional problems, SDQ ADHD |
| 4 | Parental education, Maternal mental health (GHQ), Neighbourhood deprivation (IMD) | Full scale IQ (linear term), Full scale IQ (non-linear term), Autism traits (linear term), Autism traits (non-linear term) Language development quotient, VABS - communication, VABS - daily living skills, VABS - socialisation SDQ conduct problems, SDQ Emotional problems, SDQ ADHD Parental education, Maternal mental health (GHQ), Neighbourhood deprivation (IMD) |

# Supplementary Results

### Figure S1 Flowchart

Flowchart of participant numbers at age 12 and adult assessment in the SNAP study.

### Weighted correlations

Age 12

N = 158

Age 23

N = 121

Loss to follow up

N = 37

N = 37

### Figure S2 - Correlations between age 12 predictors

Measures marked * are reversed scored so for all measures higher values indicate more challenges or worse health.


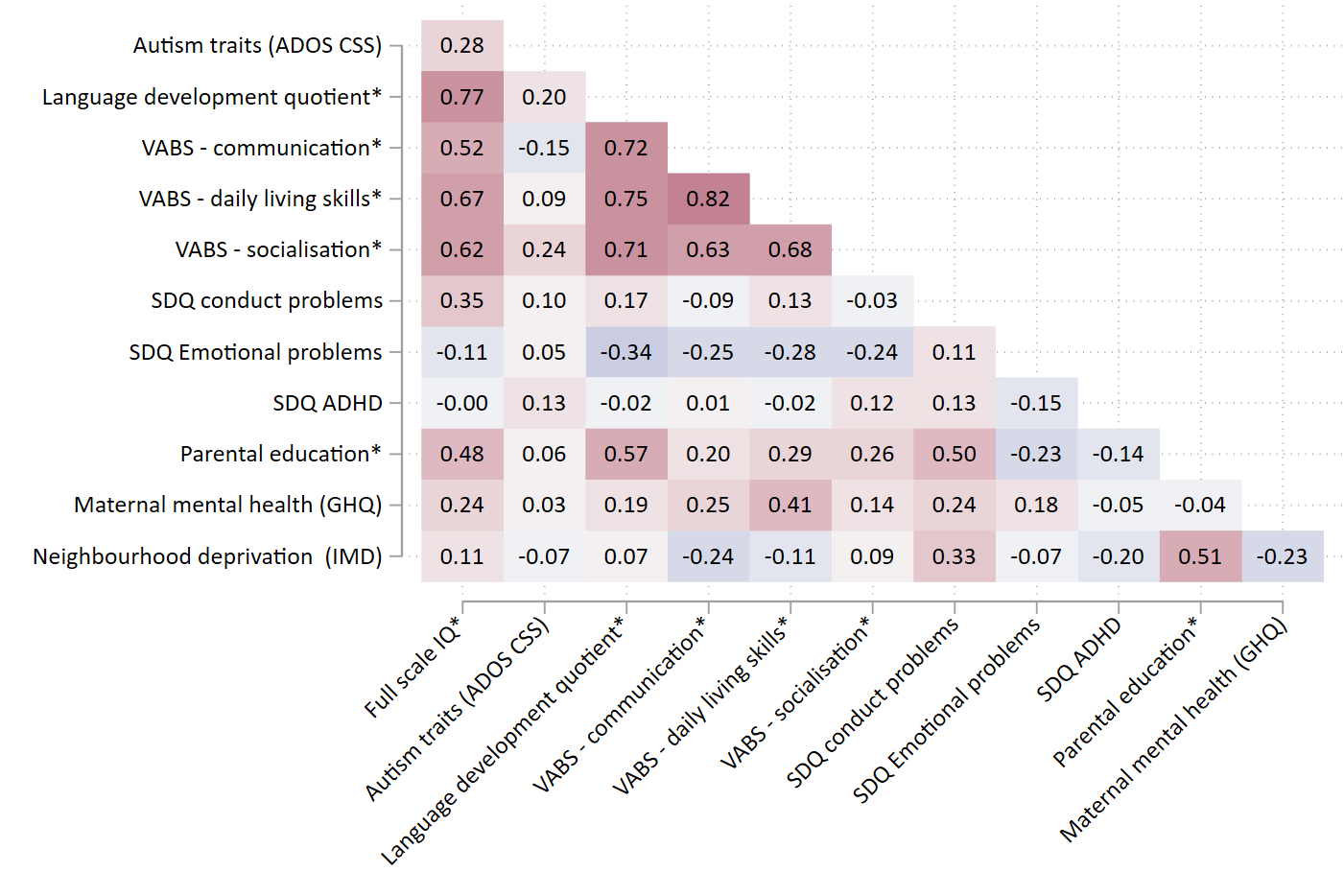


### Figure S3 - Correlations between age 23 outcomes

Measures marked * are reversed scored so for all measures higher values indicate more challenges or worse health.


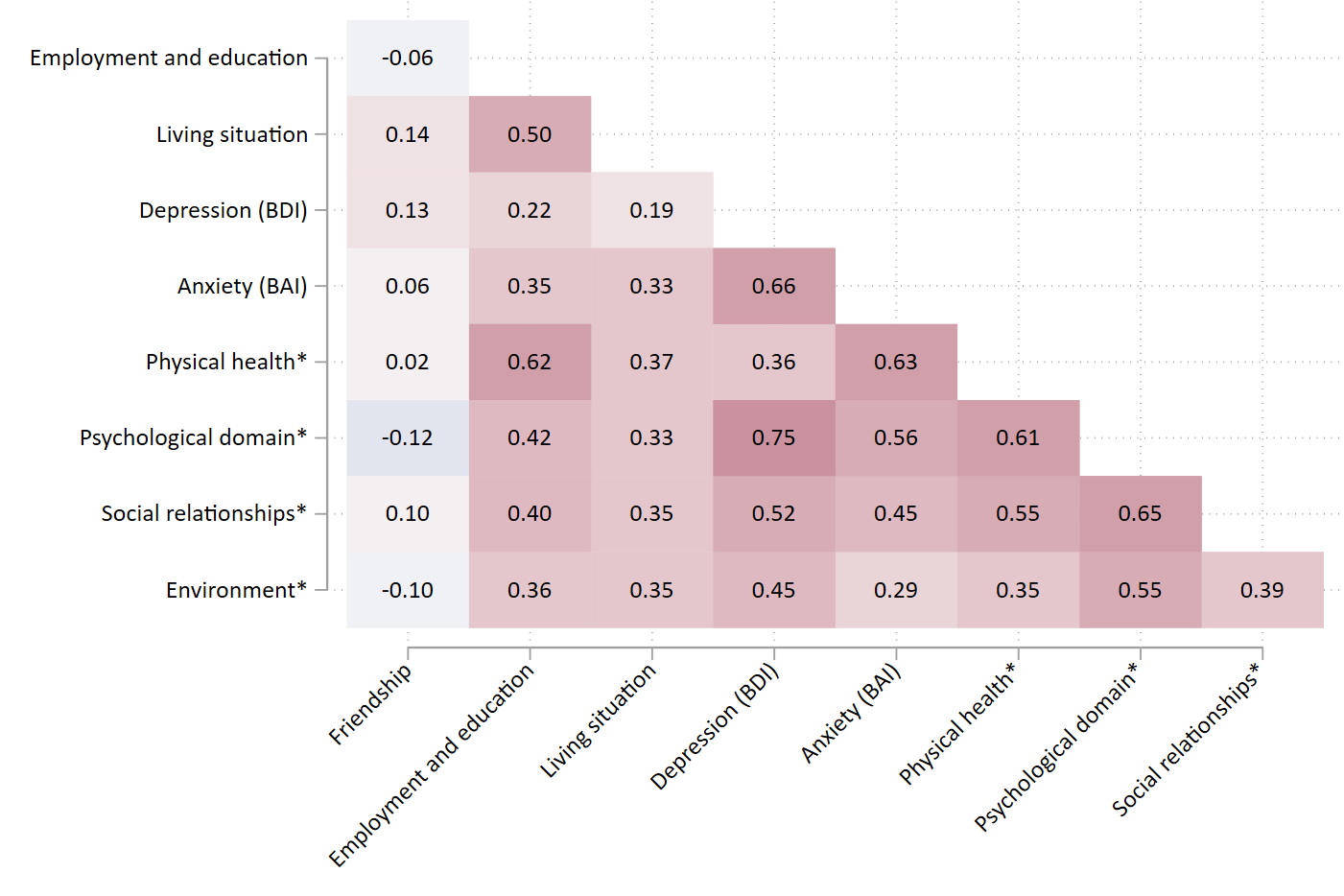


### Figure S4 - Correlations between age 12 predictors and age 23 outcomes

Measures marked * are reversed scored so for all measures higher values indicate more challenges or worse health


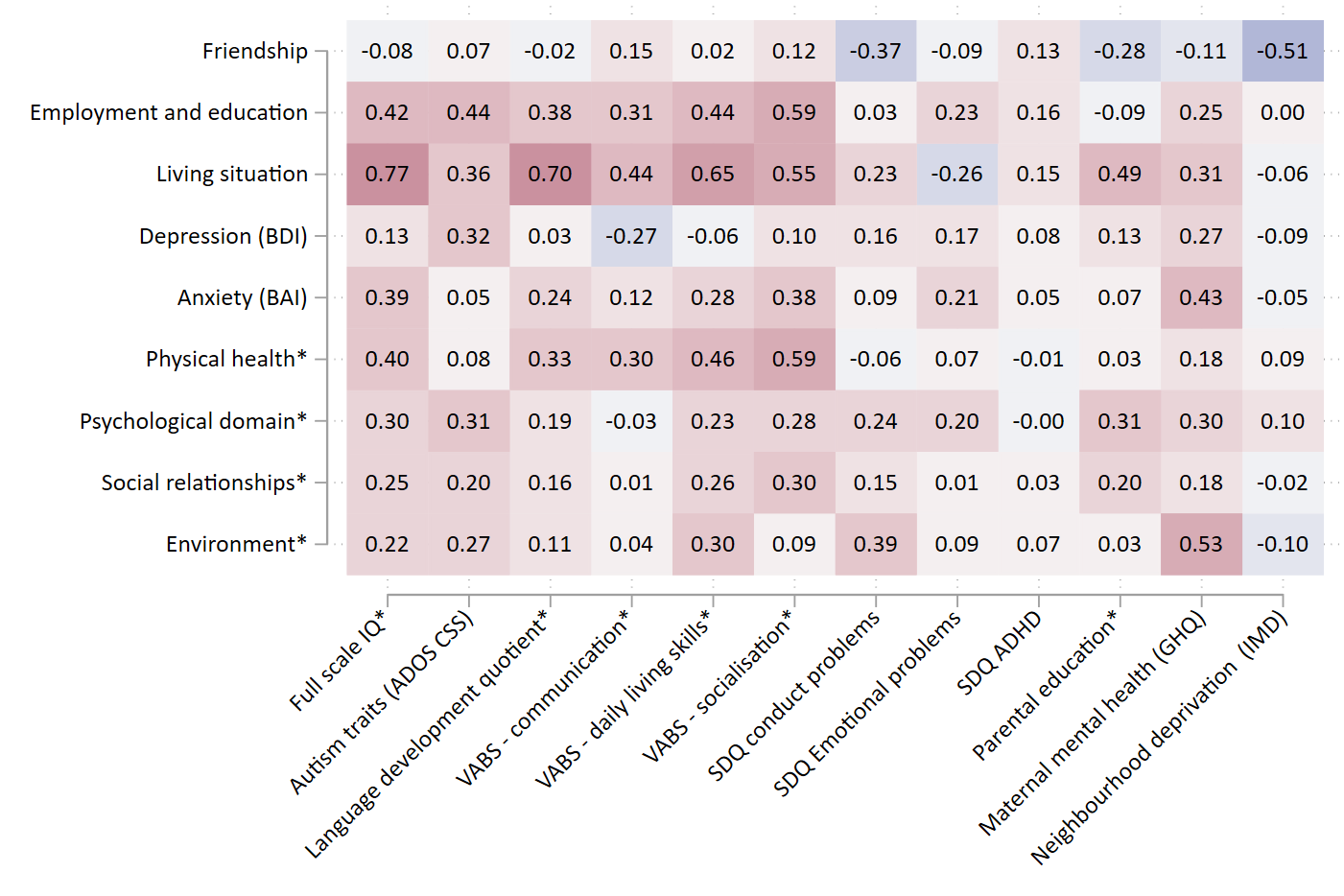


## Friendship

### Table S5 - Estimates, p-values for sets are from joint wald tests of all variables in sets.

|  | **Odds ratio (95% CI) p-value** | | | |
| --- | --- | --- | --- | --- |
| **Predictor** | **Analysis 1** | **Analysis 2** | **Analysis 3** | **Analysis 4** |
| ***Set 1*** | ***p=0.31*** | ***p=0.28*** | ***p=0.28*** | ***p=0.67*** |
| Autism traits (ADOS CSS) | p=0.14 | p=0.14 | p=0.18 | p=0.40 |
| Autism traits (linear term) | 0.686 (0.470-1.003) p=0.052 | 0.63 (0.34-1.15) p=0.13 | 0.67 (0.36-1.26) p=0.21 | 0.69 (0.40-1.18) p=0.18 |
| Autism traits (non-linear term) | 1.9 (1.0-3.6) p=0.053 | 2.5 (1.0-6.6) p=0.06 | 2.2 (0.9-5.8) p=0.10 | 1.8 (0.8-4.3) p=0.19 |
| Full scale IQ | p=0.15 | p=0.28 | p=0.52 | p=0.95 |
| Full scale IQ (linear term) | 0.958 (0.913-1.005) p=0.08 | 0.99 (0.93-1.06) p=0.84 | 0.99 (0.92-1.06) p=0.78 | 1.00 (0.94-1.07) p=0.99 |
| Full scale IQ (non-linear term) | 1.07 (1.00-1.15) p=0.052 | 1.04 (0.99-1.10) p=0.12 | 1.03 (0.98-1.09) p=0.26 | 1.01 (0.94-1.09) p=0.79 |
| ***Set 2*** |  | ***p=0.053*** | ***p=0.47*** | ***p=0.36*** |
| Language development quotient |  | 1.02 (0.93-1.12) p=0.66 | 1.01 (0.91-1.12) p=0.80 | 1.02 (0.94-1.10) p=0.69 |
| VABS - communication |  | 0.96 (0.89-1.04) p=0.31 | 0.98 (0.89-1.07) p=0.59 | 0.99 (0.93-1.06) p=0.87 |
| VABS - daily living skills |  | 1.00 (0.94-1.07) p=0.99 | 0.99 (0.92-1.07) p=0.85 | 1.01 (0.94-1.07) p=0.85 |
| VABS - socialisation |  | 0.96 (0.92-1.01) p=0.14 | 0.97 (0.92-1.03) p=0.40 | 0.931 (0.864-1.003) p=0.06 |
| ***Set 3*** |  |  | ***p=0.59*** | ***p=0.94*** |
| SDQ conduct problems |  |  | 0.81 (0.58-1.14) p=0.23 | 0.92 (0.60-1.41) p=0.71 |
| SDQ Emotional problems |  |  | 0.98 (0.78-1.21) p=0.83 | 0.96 (0.76-1.22) p=0.76 |
| SDQ ADHD |  |  | 1.05 (0.80-1.38) p=0.74 | 0.96 (0.71-1.32) p=0.82 |
| ***Set 4*** |  |  |  | ***p=0.21*** |
| Maternal mental health (GHQ) |  |  |  | 0.95 (0.85-1.06) p=0.36 |
| Neighbourhood deprivation (IMD) |  |  |  | 0.911 (0.833-0.997) p=0.042 |
| Parental education |  |  |  | 0.72 (0.19-2.77) p=0.63 |

## Employment and education

### Table S6 - Estimates, p-values for sets are from joint wald tests of all variables in sets.

|  | **Odds ratio (95% CI) p-value** | | | |
| --- | --- | --- | --- | --- |
| **Predictor** | **Analysis 1** | **Analysis 2** | **Analysis 3** | **Analysis 4** |
| ***Set 1*** | ***p=0.012*** | ***p=0.35*** | ***p=0.44*** | ***p=0.17*** |
| Autism traits (ADOS CSS) | p=0.11 | p=0.27 | p=0.21 | p=0.06 |
| Autism traits (linear term) | 1.6 (0.9-2.7) p=0.11 | 1.39 (0.77-2.51) p=0.28 | 1.38 (0.79-2.42) p=0.26 | 1.32 (0.76-2.29) p=0.33 |
| Autism traits (non-linear term) | 0.72 (0.35-1.47) p=0.37 | 0.92 (0.44-1.90) p=0.81 | 0.85 (0.40-1.84) p=0.69 | 1.17 (0.53-2.58) p=0.70 |
| Full scale IQ | p=0.37 | p=0.76 | p=0.87 | p=0.57 |
| Full scale IQ (linear term) | 0.97 (0.92-1.03) p=0.31 | 1.01 (0.94-1.08) p=0.76 | 0.99 (0.93-1.06) p=0.73 | 0.95 (0.86-1.05) p=0.30 |
| Full scale IQ (non-linear term) | 1.01 (0.95-1.08) p=0.73 | 0.97 (0.88-1.07) p=0.53 | 1.00 (0.90-1.11) p=0.96 | 1.05 (0.93-1.19) p=0.40 |
| ***Set 2*** |  | ***p=0.056*** | ***p=0.10*** | ***p=0.06*** |
| Language development quotient |  | 1.04 (0.99-1.09) p=0.13 | 1.00 (0.94-1.06) p=0.95 | 0.98 (0.90-1.07) p=0.66 |
| VABS - communication |  | 0.99 (0.93-1.04) p=0.60 | 1.02 (0.97-1.08) p=0.49 | 0.99 (0.93-1.05) p=0.75 |
| VABS - daily living skills |  | 0.98 (0.92-1.05) p=0.57 | 0.96 (0.90-1.02) p=0.18 | 0.95 (0.89-1.02) p=0.14 |
| VABS - socialisation |  | 0.91 (0.84-0.99) p=0.024 | 0.926 (0.856-1.001) p=0.054 | 0.95 (0.87-1.04) p=0.27 |
| ***Set 3*** |  |  | ***p=0.07*** | ***p=0.08*** |
| SDQ conduct problems |  |  | 0.84 (0.62-1.15) p=0.28 | 0.78 (0.49-1.24) p=0.29 |
| SDQ Emotional problems |  |  | 1.6 (1.1-2.1) p=0.007 | 1.8 (1.1-2.7) p=0.010 |
| SDQ ADHD |  |  | 1.23 (0.78-1.94) p=0.37 | 1.36 (0.82-2.24) p=0.23 |
| ***Set 4*** |  |  |  | ***p=0.08*** |
| Maternal mental health (GHQ) |  |  |  | 1.00 (0.85-1.17) p=1.00 |
| Neighbourhood deprivation (IMD) |  |  |  | 1.09 (1.01-1.17) p=0.027 |
| Parental education |  |  |  | 7.3 (1.0-54.0) p=0.052 |

### Figure S5 – Predicted probability and 95% confidence intervals of competitive employment and education from a model including IQ, ADOS CSS an interaction term between IQ and ADOS CSS at ADOS CSS of 3 and 8.

## Living situation

### Table S6 - Estimates, p-values for sets are from joint wald tests of all variables in sets.

|  | **Odds ratio (95% CI) p-value** | | | |
| --- | --- | --- | --- | --- |
| **Predictor** | **Analysis 1** | **Analysis 2** | **Analysis 3** | **Analysis 4** |
| ***Set 1*** | ***p=0.0003*** | ***p=0.11*** | ***p=0.044*** | ***p=0.003*** |
| Autism traits (ADOS CSS) | p=0.18 | p=0.16 | p=0.41 | p=0.31 |
| Autism traits (linear term) | 1.14 (0.78-1.68) p=0.49 | 0.96 (0.61-1.50) p=0.84 | 1.06 (0.69-1.63) p=0.79 | 1.17 (0.73-1.88) p=0.51 |
| Autism traits (non-linear term) | 1.06 (0.61-1.86) p=0.83 | 1.39 (0.72-2.67) p=0.32 | 1.13 (0.60-2.13) p=0.70 | 1.00 (0.50-2.00) p=0.99 |
| Full scale IQ | p=<0.0001 | p=0.024 | p=0.008 | p=0.0004 |
| Full scale IQ (linear term) | 0.863 (0.744-1.002) p=0.053 | 0.90 (0.80-1.01) p=0.08 | 0.90 (0.82-0.98) p=0.020 | 0.88 (0.81-0.94) p=0.0005 |
| Full scale IQ (non-linear term) | 1.08 (0.92-1.27) p=0.36 | 1.06 (0.93-1.21) p=0.41 | 1.05 (0.94-1.17) p=0.43 | 1.07 (0.97-1.19) p=0.18 |
| ***Set 2*** |  | ***p=0.011*** | ***p=0.037*** | ***p=0.29*** |
| Language development quotient |  | 0.962 (0.920-1.005) p=0.09 | 0.95 (0.89-1.01) p=0.12 | 0.96 (0.91-1.02) p=0.23 |
| VABS - communication |  | 1.03 (0.98-1.09) p=0.19 | 1.05 (0.99-1.11) p=0.14 | 1.05 (0.98-1.12) p=0.19 |
| VABS - daily living skills |  | 0.94 (0.89-0.99) p=0.016 | 0.93 (0.89-0.98) p=0.006 | 0.950 (0.902-1.001) p=0.053 |
| VABS - socialisation |  | 1.02 (0.95-1.10) p=0.52 | 1.05 (0.98-1.13) p=0.19 | 1.04 (0.97-1.12) p=0.22 |
| ***Set 3*** |  |  | ***p=0.30*** | ***p=0.10*** |
| SDQ conduct problems |  |  | 0.88 (0.63-1.23) p=0.46 | 0.79 (0.58-1.08) p=0.14 |
| SDQ Emotional problems |  |  | 0.96 (0.76-1.23) p=0.76 | 0.89 (0.67-1.18) p=0.42 |
| SDQ ADHD |  |  | 1.41 (0.95-2.09) p=0.08 | 1.42 (1.01-1.98) p=0.041 |
| ***Set 4*** |  |  |  | ***p=0.032*** |
| Maternal mental health (GHQ) |  |  |  | 1.12 (1.01-1.25) p=0.034 |
| Neighbourhood deprivation (IMD) |  |  |  | 0.98 (0.94-1.03) p=0.44 |
| Parental education |  |  |  | 0.2 (0.0-1.0) p=0.045 |

## Physical health

### Table S7 - Estimates, p-values for sets are from joint wald tests of all variables in sets.

|  | **Beta (95% CI) p-value** | | | |
| --- | --- | --- | --- | --- |
| **Predictor** | **Analysis 1** | **Analysis 2** | **Analysis 3** | **Analysis 4** |
| ***Set 1*** | ***p=0.20*** | ***p=0.42*** | ***p=0.10*** | ***p=0.16*** |
| Autism traits (ADOS CSS) | p=0.84 | p=0.29 | p=0.049 | p=0.11 |
| Autism traits (linear term) | -0.5 (-3.7-2.6) p=0.74 | 1.7 (-1.1-4.4) p=0.23 | 1.7 (-0.8-4.2) p=0.18 | 1.7 (-0.7-4.2) p=0.17 |
| Autism traits (non-linear term) | 1.0 (-3.2-5.2) p=0.63 | -1.3 (-4.8-2.3) p=0.47 | -0.7 (-4.4-2.9) p=0.69 | -1.0 (-4.5-2.5) p=0.56 |
| Full scale IQ | p=0.055 | p=0.50 | p=0.26 | p=0.22 |
| Full scale IQ (linear term) | 0.6 (-0.0-1.1) p=0.054 | 0.26 (-0.30-0.83) p=0.36 | 0.33 (-0.10-0.76) p=0.13 | 0.35 (-0.07-0.77) p=0.10 |
| Full scale IQ (non-linear term) | -0.47 (-1.12-0.18) p=0.16 | -0.18 (-0.82-0.45) p=0.57 | -0.27 (-0.78-0.25) p=0.31 | -0.29 (-0.80-0.21) p=0.25 |
| ***Set 2*** |  | ***p=0.002*** | ***p=<0.0001*** | ***p=0.0006*** |
| Language development quotient |  | -0.20 (-0.45-0.06) p=0.13 | -0.00 (-0.27-0.27) p=0.99 | -0.01 (-0.31-0.30) p=0.96 |
| VABS - communication |  | -0.29 (-0.55--0.03) p=0.032 | -0.48 (-0.74--0.22) p=0.0003 | -0.44 (-0.74--0.14) p=0.005 |
| VABS - daily living skills |  | 0.31 (0.05-0.56) p=0.019 | 0.42 (0.18-0.67) p=0.0010 | 0.43 (0.17-0.70) p=0.001 |
| VABS - socialisation |  | 0.7 (0.2-1.2) p=0.004 | 0.6 (0.2-1.1) p=0.004 | 0.6 (0.1-1.0) p=0.010 |
| ***Set 3*** |  |  | ***p=0.007*** | ***p=0.002*** |
| SDQ conduct problems |  |  | 1.8 (0.1-3.5) p=0.038 | 1.9 (0.0-3.8) p=0.045 |
| SDQ Emotional problems |  |  | -1.8 (-3.1--0.4) p=0.009 | -1.8 (-3.1--0.5) p=0.006 |
| SDQ ADHD |  |  | -0.39 (-2.12-1.35) p=0.66 | -0.5 (-2.3-1.3) p=0.57 |
| ***Set 4*** |  |  |  | ***p=0.80*** |
| Maternal mental health (GHQ) |  |  |  | -0.02 (-0.58-0.55) p=0.96 |
| Neighbourhood deprivation (IMD) |  |  |  | -0.13 (-0.38-0.12) p=0.32 |
| Parental education |  |  |  | -1.3 (-9.0-6.4) p=0.74 |

## Psychological domain

### Table S8 - Estimates, p-values for sets are from joint wald tests of all variables in sets.

|  | **Beta (95% CI) p-value** | | | |
| --- | --- | --- | --- | --- |
| **Predictor** | **Analysis 1** | **Analysis 2** | **Analysis 3** | **Analysis 4** |
| ***Set 1*** | ***p=0.06*** | ***p=0.33*** | ***p=0.78*** | ***p=0.77*** |
| Autism traits (ADOS CSS) | p=0.12 | p=0.78 | p=0.96 | p=0.90 |
| Autism traits (linear term) | -2.1 (-5.1-0.9) p=0.17 | -0.6 (-3.5-2.3) p=0.70 | 0.05 (-2.45-2.56) p=0.97 | -0.03 (-2.45-2.38) p=0.98 |
| Autism traits (non-linear term) | 1.5 (-3.5-6.5) p=0.54 | 0.31 (-4.02-4.63) p=0.89 | -0.35 (-4.62-3.92) p=0.87 | -0.34 (-4.18-3.51) p=0.86 |
| Full scale IQ | p=0.10 | p=0.12 | p=0.47 | p=0.55 |
| Full scale IQ (linear term) | -0.08 (-0.42-0.26) p=0.64 | -0.16 (-0.56-0.23) p=0.42 | -0.15 (-0.57-0.27) p=0.48 | -0.12 (-0.53-0.28) p=0.54 |
| Full scale IQ (non-linear term) | 0.33 (-0.15-0.81) p=0.17 | 0.41 (-0.06-0.88) p=0.09 | 0.29 (-0.21-0.80) p=0.25 | 0.25 (-0.24-0.75) p=0.31 |
| ***Set 2*** |  | ***p=0.007*** | ***p=0.003*** | ***p=0.009*** |
| Language development quotient |  | -0.07 (-0.30-0.16) p=0.56 | 0.06 (-0.21-0.32) p=0.68 | -0.01 (-0.28-0.26) p=0.92 |
| VABS - communication |  | -0.36 (-0.67--0.06) p=0.020 | -0.43 (-0.74--0.13) p=0.006 | -0.41 (-0.72--0.10) p=0.011 |
| VABS - daily living skills |  | 0.29 (0.09-0.49) p=0.006 | 0.35 (0.14-0.56) p=0.001 | 0.30 (0.09-0.50) p=0.005 |
| VABS - socialisation |  | 0.35 (-0.01-0.71) p=0.06 | 0.33 (-0.04-0.71) p=0.08 | 0.36 (-0.05-0.77) p=0.09 |
| ***Set 3*** |  |  | ***p=0.18*** | ***p=0.16*** |
| SDQ conduct problems |  |  | -0.10 (-1.82-1.62) p=0.91 | 0.44 (-1.35-2.22) p=0.63 |
| SDQ Emotional problems |  |  | -1.4 (-2.7--0.1) p=0.032 | -1.3 (-2.5--0.1) p=0.029 |
| SDQ ADHD |  |  | -0.33 (-1.97-1.31) p=0.69 | -0.46 (-2.09-1.16) p=0.57 |
| ***Set 4*** |  |  |  | ***p=0.17*** |
| Maternal mental health (GHQ) |  |  |  | -0.41 (-0.87-0.06) p=0.09 |
| Neighbourhood deprivation (IMD) |  |  |  | 0.02 (-0.27-0.32) p=0.87 |
| Parental education |  |  |  | 6.3 (-1.9-14.5) p=0.13 |

## Social relationships

### Table S9 - Estimates, p-values for sets are from joint wald tests of all variables in sets.

|  | **Beta (95% CI) p-value** | | | |
| --- | --- | --- | --- | --- |
| **Predictor** | **Analysis 1** | **Analysis 2** | **Analysis 3** | **Analysis 4** |
| ***Set 1*** | ***p=0.06*** | ***p=0.33*** | ***p=0.78*** | ***p=0.77*** |
| Autism traits (ADOS CSS) | p=0.12 | p=0.78 | p=0.96 | p=0.90 |
| Autism traits (linear term) | -2.1 (-5.1-0.9) p=0.17 | -0.6 (-3.5-2.3) p=0.70 | 0.05 (-2.45-2.56) p=0.97 | -0.03 (-2.45-2.38) p=0.98 |
| Autism traits (non-linear term) | 1.5 (-3.5-6.5) p=0.54 | 0.31 (-4.02-4.63) p=0.89 | -0.35 (-4.62-3.92) p=0.87 | -0.34 (-4.18-3.51) p=0.86 |
| Full scale IQ | p=0.10 | p=0.12 | p=0.47 | p=0.55 |
| Full scale IQ (linear term) | -0.08 (-0.42-0.26) p=0.64 | -0.16 (-0.56-0.23) p=0.42 | -0.15 (-0.57-0.27) p=0.48 | -0.12 (-0.53-0.28) p=0.54 |
| Full scale IQ (non-linear term) | 0.33 (-0.15-0.81) p=0.17 | 0.41 (-0.06-0.88) p=0.09 | 0.29 (-0.21-0.80) p=0.25 | 0.25 (-0.24-0.75) p=0.31 |
| ***Set 2*** |  | ***p=0.007*** | ***p=0.003*** | ***p=0.009*** |
| Language development quotient |  | -0.07 (-0.30-0.16) p=0.56 | 0.06 (-0.21-0.32) p=0.68 | -0.01 (-0.28-0.26) p=0.92 |
| VABS - communication |  | -0.36 (-0.67--0.06) p=0.020 | -0.43 (-0.74--0.13) p=0.006 | -0.41 (-0.72--0.10) p=0.011 |
| VABS - daily living skills |  | 0.29 (0.09-0.49) p=0.006 | 0.35 (0.14-0.56) p=0.001 | 0.30 (0.09-0.50) p=0.005 |
| VABS - socialisation |  | 0.35 (-0.01-0.71) p=0.06 | 0.33 (-0.04-0.71) p=0.08 | 0.36 (-0.05-0.77) p=0.09 |
| ***Set 3*** |  |  | ***p=0.18*** | ***p=0.16*** |
| SDQ conduct problems |  |  | -0.10 (-1.82-1.62) p=0.91 | 0.44 (-1.35-2.22) p=0.63 |
| SDQ Emotional problems |  |  | -1.4 (-2.7--0.1) p=0.032 | -1.3 (-2.5--0.1) p=0.029 |
| SDQ ADHD |  |  | -0.33 (-1.97-1.31) p=0.69 | -0.46 (-2.09-1.16) p=0.57 |
| ***Set 4*** |  |  |  | ***p=0.17*** |
| Maternal mental health (GHQ) |  |  |  | -0.41 (-0.87-0.06) p=0.09 |
| Neighbourhood deprivation (IMD) |  |  |  | 0.02 (-0.27-0.32) p=0.87 |
| Parental education |  |  |  | 6.3 (-1.9-14.5) p=0.13 |

## Environment

### Table S10 - Estimates, p-values for sets are from joint wald tests of all variables in sets.

|  | **Beta (95% CI) p-value** | | | |
| --- | --- | --- | --- | --- |
| **Predictor** | **Analysis 1** | **Analysis 2** | **Analysis 3** | **Analysis 4** |
| ***Set 1*** | ***p=0.14*** | ***p=0.26*** | ***p=0.28*** | ***p=0.27*** |
| Autism traits (ADOS CSS) | p=0.16 | p=0.19 | p=0.15 | p=0.11 |
| Autism traits (linear term) | -2.5 (-5.4-0.5) p=0.10 | -1.6 (-4.3-1.1) p=0.24 | -0.7 (-3.4-1.9) p=0.59 | -1.1 (-3.4-1.2) p=0.34 |
| Autism traits (non-linear term) | 2.3 (-1.3-5.9) p=0.21 | 1.1 (-2.3-4.5) p=0.52 | -0.35 (-3.88-3.18) p=0.85 | 0.34 (-3.00-3.68) p=0.84 |
| Full scale IQ | p=0.83 | p=0.75 | p=0.36 | p=0.62 |
| Full scale IQ (linear term) | 0.08 (-0.28-0.44) p=0.67 | 0.12 (-0.24-0.48) p=0.53 | 0.07 (-0.22-0.36) p=0.64 | 0.05 (-0.22-0.32) p=0.70 |
| Full scale IQ (non-linear term) | -0.05 (-0.47-0.38) p=0.83 | -0.08 (-0.47-0.31) p=0.68 | -0.18 (-0.48-0.12) p=0.24 | -0.12 (-0.41-0.16) p=0.40 |
| ***Set 2*** |  | ***p=0.034*** | ***p=0.051*** | ***p=0.69*** |
| Language development quotient |  | -0.12 (-0.33-0.10) p=0.28 | -0.10 (-0.31-0.11) p=0.33 | -0.08 (-0.29-0.13) p=0.44 |
| VABS - communication |  | -0.16 (-0.37-0.06) p=0.16 | -0.08 (-0.30-0.15) p=0.49 | -0.08 (-0.33-0.18) p=0.55 |
| VABS - daily living skills |  | 0.36 (0.13-0.60) p=0.003 | 0.35 (0.13-0.58) p=0.003 | 0.22 (-0.08-0.51) p=0.15 |
| VABS - socialisation |  | -0.19 (-0.59-0.21) p=0.35 | -0.11 (-0.46-0.24) p=0.53 | -0.07 (-0.35-0.22) p=0.65 |
| ***Set 3*** |  |  | ***p=0.008*** | ***p=0.017*** |
| SDQ conduct problems |  |  | -1.8 (-3.0--0.6) p=0.004 | -1.6 (-2.7--0.5) p=0.005 |
| SDQ Emotional problems |  |  | -0.5 (-1.6-0.5) p=0.30 | -0.06 (-1.02-0.90) p=0.91 |
| SDQ ADHD |  |  | -0.10 (-1.66-1.47) p=0.90 | -0.09 (-1.37-1.19) p=0.89 |
| ***Set 4*** |  |  |  | ***p=0.043*** |
| Maternal mental health (GHQ) |  |  |  | -0.7 (-1.3--0.0) p=0.041 |
| Neighbourhood deprivation (IMD) |  |  |  | 0.05 (-0.19-0.29) p=0.67 |
| Parental education |  |  |  | -0.7 (-7.0-5.6) p=0.82 |

## Depression symptoms (Log BDI)

### Table S11 - Estimates, p-values for sets are from joint wald tests of all variables in sets.

|  | **Beta (95% CI) p-value** | | | |
| --- | --- | --- | --- | --- |
| **Predictor** | **Analysis 1** | **Analysis 2** | **Analysis 3** | **Analysis 4** |
| ***Set 1*** | ***p=0.28*** | ***p=0.48*** | ***p=0.44*** | ***p=0.22*** |
| Autism traits (ADOS CSS) | p=0.35 | p=0.61 | p=0.39 | p=0.54 |
| Autism traits (linear term) | 0.12 (-0.28-0.52) p=0.56 | -0.12 (-0.46-0.22) p=0.48 | -0.20 (-0.52-0.13) p=0.23 | -0.15 (-0.42-0.13) p=0.28 |
| Autism traits (non-linear term) | 0.06 (-0.61-0.73) p=0.86 | 0.30 (-0.30-0.90) p=0.33 | 0.40 (-0.18-0.98) p=0.17 | 0.24 (-0.19-0.68) p=0.27 |
| Full scale IQ | p=0.73 | p=0.50 | p=0.49 | p=0.22 |
| Full scale IQ (linear term) | 0.00 (-0.04-0.04) p=0.88 | 0.02 (-0.02-0.06) p=0.30 | 0.02 (-0.02-0.06) p=0.25 | 0.03 (-0.00-0.07) p=0.09 |
| Full scale IQ (non-linear term) | -0.02 (-0.07-0.04) p=0.58 | -0.03 (-0.08-0.02) p=0.25 | -0.02 (-0.07-0.03) p=0.47 | -0.034 (-0.077-0.009) p=0.12 |
| ***Set 2*** |  | ***p=<0.0001*** | ***p=<0.0001*** | ***p=0.001*** |
| Language development quotient |  | 0.01 (-0.02-0.03) p=0.64 | -0.00 (-0.02-0.02) p=0.93 | -0.01 (-0.03-0.02) p=0.57 |
| VABS - communication |  | 0.05 (-0.00-0.10) p=0.06 | 0.05 (0.00-0.09) p=0.032 | 0.06 (0.03-0.10) p=0.0008 |
| VABS - daily living skills |  | -0.05 (-0.08--0.03) p=<0.0001 | -0.06 (-0.08--0.03) p=<0.0001 | -0.04 (-0.07--0.01) p=0.004 |
| VABS - socialisation |  | -0.032 (-0.072-0.008) p=0.11 | -0.035 (-0.081-0.010) p=0.12 | -0.06 (-0.10--0.01) p=0.013 |
| ***Set 3*** |  |  | ***p=0.28*** | ***p=0.52*** |
| SDQ conduct problems |  |  | 0.09 (-0.09-0.27) p=0.33 | 0.11 (-0.06-0.28) p=0.20 |
| SDQ Emotional problems |  |  | 0.10 (-0.07-0.27) p=0.25 | 0.03 (-0.10-0.15) p=0.64 |
| SDQ ADHD |  |  | 0.00 (-0.17-0.18) p=0.96 | -0.04 (-0.20-0.12) p=0.65 |
| ***Set 4*** |  |  |  | ***p=0.004*** |
| Maternal mental health (GHQ) |  |  |  | 0.06 (0.01-0.12) p=0.013 |
| Neighbourhood deprivation (IMD) |  |  |  | -0.043 (-0.083--0.003) p=0.037 |
| Parental education |  |  |  | -0.17 (-1.06-0.72) p=0.71 |

## Anxiety symptoms

### Table S12 - Estimates, p-values for sets are from joint wald tests of all variables in sets.

|  | **RR (95% CI) p-value** | | | |
| --- | --- | --- | --- | --- |
| **Predictor** | **Analysis 1** | **Analysis 2** | **Analysis 3** | **Analysis 4** |
| ***Set 1*** | ***p=0.005*** | ***p=<0.0001*** | ***p=0.0001*** | ***p=0.0001*** |
| Autism traits (ADOS CSS) | p=0.54 | p=0.009 | p=0.005 | p=0.010 |
| Autism traits (linear term) | 0.897 (0.733-1.098) p=0.29 | 0.767 (0.636-0.925) p=0.006 | 0.754 (0.631-0.902) p=0.002 | 0.785 (0.666-0.925) p=0.004 |
| Autism traits (non-linear term) | 1.115 (0.824-1.508) p=0.48 | 1.270 (0.923-1.746) p=0.14 | 1.304 (1.002-1.699) p=0.049 | 1.207 (0.961-1.515) p=0.11 |
| Full scale IQ | p=0.002 | p=0.009 | p=0.022 | p=0.005 |
| Full scale IQ (linear term) | 0.985 (0.969-1.001) p=0.07 | 0.993 (0.967-1.019) p=0.59 | 0.990 (0.968-1.013) p=0.41 | 0.990 (0.964-1.015) p=0.43 |
| Full scale IQ (non-linear term) | 0.988 (0.961-1.017) p=0.41 | 0.975 (0.944-1.006) p=0.11 | 0.983 (0.952-1.014) p=0.28 | 0.981 (0.949-1.015) p=0.27 |
| ***Set 2*** |  | ***p=<0.0001*** | ***p=0.007*** | ***p=0.024*** |
| Language development quotient |  | 1.010 (0.990-1.031) p=0.33 | 0.998 (0.978-1.019) p=0.85 | 0.996 (0.976-1.017) p=0.70 |
| VABS - communication |  | 1.031 (1.002-1.061) p=0.036 | 1.038 (1.013-1.064) p=0.002 | 1.041 (1.015-1.067) p=0.002 |
| VABS - daily living skills |  | 0.970 (0.948-0.992) p=0.009 | 0.965 (0.944-0.987) p=0.002 | 0.974 (0.952-0.996) p=0.022 |
| VABS - socialisation |  | 0.968 (0.946-0.990) p=0.004 | 0.976 (0.952-1.000) p=0.055 | 0.970 (0.939-1.002) p=0.06 |
| ***Set 3*** |  |  | ***p=0.07*** | ***p=0.28*** |
| SDQ conduct problems |  |  | 0.952 (0.850-1.066) p=0.39 | 0.941 (0.824-1.075) p=0.37 |
| SDQ Emotional problems |  |  | 1.119 (1.022-1.225) p=0.015 | 1.083 (0.993-1.182) p=0.07 |
| SDQ ADHD |  |  | 1.043 (0.924-1.178) p=0.50 | 1.035 (0.912-1.175) p=0.59 |
| ***Set 4*** |  |  |  | ***p=0.047*** |
| Maternal mental health (GHQ) |  |  |  | 1.039 (1.003-1.076) p=0.035 |
| Neighbourhood deprivation (IMD) |  |  |  | 0.990 (0.966-1.014) p=0.39 |
| Parental education |  |  |  | 0.920 (0.578-1.465) p=0.73 |

## Attrition Analysis

### Table S13 - Attrition analysis

Descriptive statistics comparing baseline variables between those who completed adult follow up and those lost to follow up. The association of baseline variables with loss to follow up is from a logistic regression model with loss to follow up as outcome and the respective baseline variable as a single predictor, without taking into account sampling weights.

| **Age 12 Variable** | **N**  **enrolled** | **Age 12**  **mean (sd)/n %** | **N at**  **age 23 follow up** | **Mean (sd)/% for those followed up at age 23** | | **Association of baseline variable with loss to follow up** | |
| --- | --- | --- | --- | --- | --- | --- | --- |
|  |  |  |  | **Weighted** | **Unweighted** | **OR (95% CI)** | **p-value** |
| Age (years) | 158 | 11.6 (1.0) | 121 | 11.9 (0.9) | 11.7 (0.9) | 1.5 (1.0 to 2.2) | 0.074 |
| Male - n (%) | 158 | 142 (89.9%) | 121 | 83% | 106 (87.6%) | 5.1 (0.6 to 39.9) | 0.121 |
| Autism traits (ADOS CSS) | 154 | 6 (3) | 121 | 6 (3) | 6 (3) | 1.0 (0.9 to 1.2) | 0.645 |
| Full scale IQ | 156 | 72.2 (24.5) | 121 | 70.0 (23.4) | 73.2 (24.4) | 1.0 (1.0 to 1.0) | 0.373 |
| Language development quotient | 147 | 54.9 (24.9) | 117 | 52.8 (21.7) | 55.0 (25.2) | 1.0 (1.0 to 1.0) | 0.914 |
| ***Vineland adaptive behaviour scales (standard scores)*** | | | | | |  |  |
| VABS - composite | 141 | 45.4 (16.6) | 112 | 49.9 (16.0) | 46.0 (16.8) | 1.0 (1.0 to 1.0) | 0.369 |
| VABS - communication | 141 | 56.8 (23.3) | 112 | 60.3 (22.0) | 57.5 (23.9) | 1.0 (1.0 to 1.0) | 0.510 |
| VABS - daily living skills | 141 | 42.8 (20.7) | 112 | 50.9 (22.4) | 43.5 (20.4) | 1.0 (1.0 to 1.0) | 0.377 |
| VABS - socialisation | 141 | 48.9 (14.3) | 112 | 50.7 (13.4) | 49.0 (14.2) | 1.0 (1.0 to 1.0) | 0.919 |
| ***Strengths and difficulties questionnaire*** | | | | | |  |  |
| SDQ total difficulties | 146 | 21.2 (6.0) | 114 | 22.1 (4.6) | 20.9 (5.9) | 1.0 (0.9 to 1.0) | 0.194 |
| SDQ conduct problems | 146 | 3.4 (2.4) | 114 | 3.9 (2.3) | 3.4 (2.2) | 1.0 (0.8 to 1.1) | 0.653 |
| SDQ ADHD | 146 | 7.6 (2.4) | 114 | 7.9 (2.1) | 7.4 (2.4) | 0.9 (0.7 to 1.1) | 0.153 |
| SDQ Emotional problems | 146 | 4.6 (2.7) | 114 | 4.5 (2.8) | 4.6 (2.7) | 1.0 (0.9 to 1.1) | 0.897 |
| ***Parental and neighbourhood characteristics*** | | | | | |  |  |
| Parental education - n (%) | | | | | | 3.0 (1.4 to 6.4) | 0.004 |
| Up to high school diploma | 158 | 54 (34.2%) | 121 | 50% | 34 (28.1%) |  |  |
| Post high school diploma | 158 | 104 (65.8%) | 121 | 50% | 87 (71.9%) |  |  |
| Maternal mental health (GHQ) | 127 | 5.1 (6.5) | 103 | 4.2 (6.0) | 5.3 (6.7) | 1.0 (1.0 to 1.1) | 0.449 |
| Neighbourhood deprivation (IMD) | 153 | 17.1 (11.5) | 117 | 20.4 (14.1) | 15.7 (11.2) | 1.0 (0.9 to 1.0) | 0.007 |

# References

Dunn, L. M., Dunn, L. M., Whetton, C., & Burley, J. (1997). *British Picture Vocabulary Scale (2nd ed.)*. NFER-Nelson.

Howlin, P., Goode, S., Hutton, J., & Rutter, M. (2004). Adult outcome for children with autism. *Journal of Child Psychology and Psychiatry, and Allied Disciplines*, *45*(2), 212–229. https://doi.org/10.1111/j.1469-7610.2004.00215.x

Raven, J., Court, J., & Raven, J. (1990a). *Coloured Progressive Matrices*. Oxford University Press.

Raven, J., Court, J., & Raven, J. (1990b). *Standard Progressive Matrices*. Oxford University Press.

*Stata Statistical Software: Release 16*. (2019). StataCorp LLC.

White, I. R., Royston, P., & Wood, A. M. (2011). Multiple imputation using chained equations: Issues and guidance for practice. *Statistics in Medicine*, *30*(4), 377–399. https://doi.org/10.1002/sim.4067
